# Altered cofactor recruitment and nucleosome dynamics underlie bisphenol A’s impact on ERα-mediated transcriptional bursting

**DOI:** 10.1016/j.isci.2025.112864

**Published:** 2025-06-10

**Authors:** Christopher R. Day, Pelin Yaşar, Gloria Adedoyin, Brian D. Bennett, Carson C. Chow, Joseph Rodriguez

**Affiliations:** 1 Epigenetics and RNA Biology Laboratory, National Institute of Environmental Health Sciences, Research Triangle Park, Durham, NC 27709, USA; 2 Integrative Bioinformatics Support Group, Biostatistics and Computational Biology Branch, National Institute of Environmental Health Sciences, Research Triangle Park, Durham, NC 27709, USA; 3 Laboratory of Biological Modeling, National Institute of Diabetes and Digestive and Kidney Diseases, NIH, Bethesda, MD 20892, USA

**Keywords:** Biochemistry, Molecular biology, Molecular interaction

## Abstract

Transcription initiation at hormone-responsive gene promoters involves recruitment of numerous proteins that initiate stochastic events known as transcriptional bursts. Estrogen responsive genes are regulated by Estradiol (E2) that binds to the Estrogen Receptor-α (ERα), allowing ERα to interact with DNA regulatory elements, recruit cofactors, and initiate transcription. Here, we utilized single-molecule imaging to determine how ERα mediated transcription is altered by non-canonical ligands such as Bisphenol A (BPA). Our analysis showed that the *TFF1* gene exhibited similar burst initiation kinetics in BPA-treated cells compared to cells treated with E2. However, there was a significant reduction in the number of active alleles in the BPA-treated cells. We show that while BPA bound ERα did induce chromatin remodeling, nucleosome positioning was altered and coincided with reduced transcription factor binding. Additionally, BPA treatment impaired enhancer mediated *TFF1* bursting. Together, this demonstrates that BPA disrupts transcriptional states by altering gene specific ERα cofactor recruitment.

## Highlights

• Single-molecule imaging studies of ERα transcription with non-canonical ligands
• ERα bound by ligands initiates maximal burst initiation rates at permissive alleles
• BPA-bound ERα remodels chromatin resulting in phasic nucleosome structure
• BPA treatment impairs enhancer-mediated *TFF1* bursting

## Graphical abstract

**Figure:**
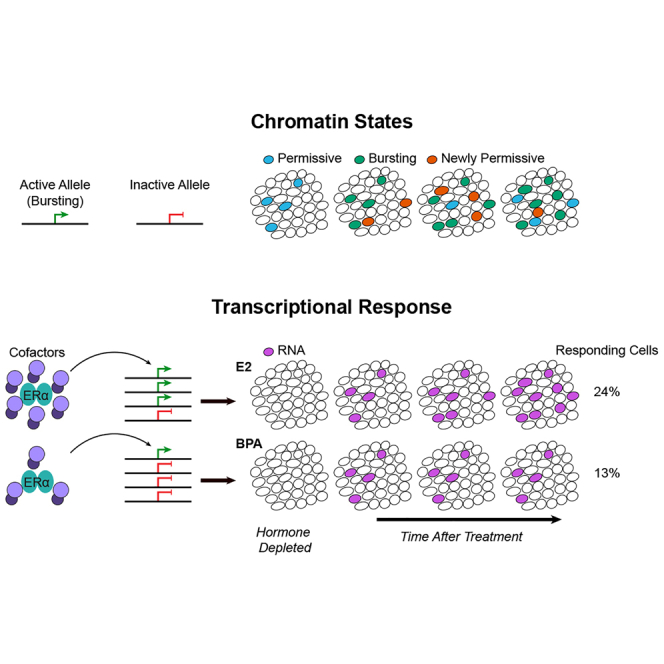

## Introduction

Transcriptional initiation requires the precise coordination of many gene regulatory proteins with specialized functions. This complex regulation at single alleles results in stochastic episodes of nascent RNA synthesis known as transcriptional bursts.^1^^,^^2^ The initiation of these bursts is controlled by the recruitment of transcription factors to regulatory elements in the enhancer and promoter regions of the gene.^3^^,^^4^ However, chromatin in these gene regulatory regions can become inaccessible to transcription factors when it is tightly wrapped around nucleosomes.^5^^,^^6^ Therefore, the remodeling of chromatin surrounding regulatory elements from nonpermissive to permissive chromatin states is a requisite step toward the initiation of a transcriptional burst.

Hormone responsive genes are typified by large-scale chromatin remodeling upon ligand stimulation.^7^^,^^8^^,^^9^ This makes study of this class of genes particularly useful for determining the relationship between chromatin states and the transcriptional state. For example, transcription of estrogen responsive genes is controlled by the primary circulating estrogen, 17-β-estradiol (E2), bound to Estrogen Receptor-α (ERα).^10^ The ligand bound ERα then binds to thousands of estrogen response elements (EREs) across the genome leading to changes in transcription of target genes.^11^^,^^12^^,^^13^ Studies of estrogen responsive genes using bulk cell assays as well as imaging of transcription in single cells have illustrated heterogeneity in hormone responses. Despite large fold changes in gene transcription observed in bulk cell assays, only a fraction of cells respond to the hormone, initiating transcriptional bursts at ERα target genes.^14^^,^^15^^,^^16^ Additionally, real-time imaging of these target genes showed that only a fraction of the alleles are transcriptionally permissive and bursting. Furthermore, as the concentration of E2 increases, both the number of bursting alleles and the frequency of burst initiation increases.^15^^,^^16^ Interestingly, the levels of active ERα within an individual cell do not appear to be responsible for this heterogeneous response.^17^ Instead, these observations indicate that alleles of ERα target genes exist in multiple chromatin states.

The binding of E2 to the ligand binding domain (LBD) of ERα causes a conformational change in the receptor into a transcriptionally active form, exposing new surfaces for protein-protein interactions,^18^^,^^19^ which are then recruited by the ERα to target gene regulatory elements. Among the proteins that interact with active ERα is the pioneer factor FOXA1.^20^ FOXA1 interacts with ERα bound to EREs to facilitate the establishment of permissive chromatin states.^21^^,^^22^^,^^23^ Other cofactors are recruited by ERα that induce chromatin remodeling, including Histone 3 Lysine 27 acetylation (H3K27ac); a modification that marks accessible chromatin.^24^^,^^25^ The deposition of K27ac is mediated by the p160 family of proteins that include the Steroid Receptor Coactivators (SRC-) 1, 2, and 3, which are pivotal cofactors in nuclear receptor transcriptional activation, including ERα signaling.^26^ Additionally, the Mediator complex, which functions to regulate the interactions between enhancers and promoters, contains an ERα interacting subunit, MED1, that facilitates interaction between the general transcription machinery and ERα.^27^ Thus, the ligand-dependent recruitment of cofactors and pioneering factors by ERα is key for transcriptional burst initiation.^28^ However, our understanding of how these factors regulate burst features such as burst initiation rates and burst size is limited.

Estrogen-mimicking chemicals known as endocrine-disrupting chemicals (EDCs) bind to the ERα LBD resulting in altered receptor protein conformations,^29^ leading to altered interactions between ERα and many of its cofactors.^30^^,^^31^ Specifically, *in vitro* assays have shown that ERα bound by Bisphenol A (BPA) results in the loss of interactions with up to 80% of assayed cofactors, including MED1 and SRC-3.^31^ This results in reduced ERα binding to chromatin and an altered transcriptional response.^29^^,^^32^^,^^33^ However, it is unknown how these effects alter bursting kinetics at individual alleles and make EDCs an ideal tool to study how limited cofactor recruitment alters ERα mediated transcriptional bursting.

Here, we use single molecule imaging of transcription to investigate how EDCs alter the ability of ERα to initiate transcriptional bursting of estrogen responsive genes in MCF-7 breast cancer cells. We find that BPA treatment maximally induces burst initiation of the E2 responsive gene *TFF1.* However, there are significantly fewer active alleles in BPA treated cell populations compared to those treated with E2. Additionally, we observe reduced recruitment of cofactors and transcription factors, including ERα to *TFF1*. Our data suggest that efficient recruitment of these factors is important for switching alleles from a transcriptionally nonpermissive state to a permissive state. Although we observed no effect on overall chromatin accessibility, we observed increased nucleosome positioning over the *TFF1* ERE indicating that some cofactors are responsible for correct nucleosome positioning. In addition to proper recruitment of ERα and cofactors, the enhancer of *TFF1* plays a key role in this state switching and does not behave synergistically with the promoter in the presence of BPA. Our work illustrates how cofactors help position nucleosomes on specific genes, which ultimately determine the extent of the single cell response.

## Results

### *TFF1* transcriptional burst frequencies are consistent between E2 and BPA treated cells

**Figure 1:**
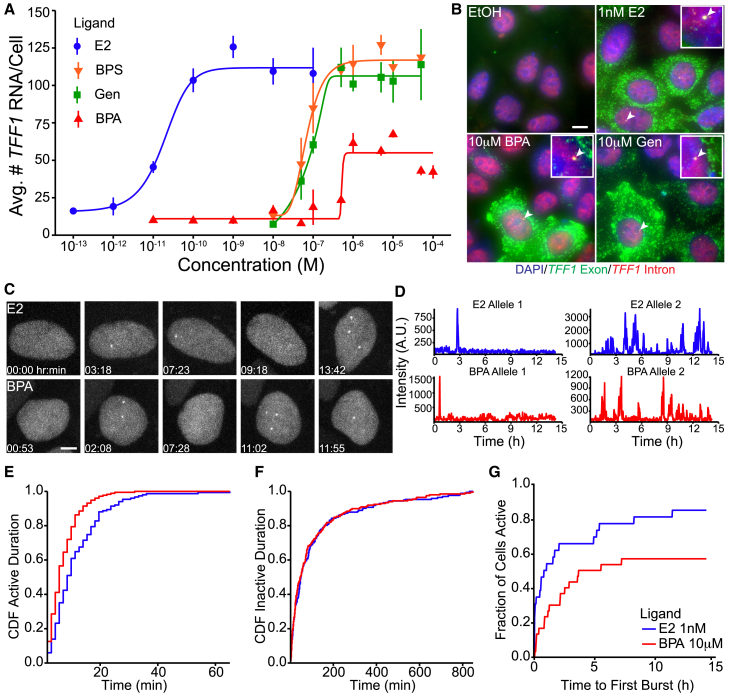
Live-cell imaging of *TFF1* transcription dynamics reveals similar burst frequencies between E2 and BPA treatment (A) Dose-dependent accumulation of *TFF1* RNA calculated from 3 smFISH replicates in response to E2, BPA, Gen, or BPS. Error bars represent standard deviation of the average number of *TFF1* RNA for each replicate. (B) *TFF1* smFISH in MCF-7 cells treated with Ethanol (EtOH), Estrogen (E2), Bisphenol S (BPS), Bisphenol-A (BPA) or Genistein (Gen), insets depict transcription sites (TS). Scale bar represents 10μm. (C) Visualization of active *TFF1-MS2* transcription in cells treated with E2 or BPA. Scale bar represents 5μm. (D) Single allele fluorescent intensity traces extracted from live-cell imaging data. Alleles from E2 treated cells (blue) or BPA treated cells (red). (E) Cumulative distribution functions (CDF) of the active durations extracted from live-cell imaging data in cells treated with E2 (blue) or BPA (red). (F) CDF of the inactive durations between bursts extracted from live-cell imaging data. Cells treated with E2 or BPA. *p*-values were calculated with Kolmogorov-Smirnov tests. (G) CDF of the fraction of active cells over time treated with either E2 or BPA, extracted from live-cell imaging.

To understand if differential recruitment of cofactors by EDC bound ERα alters the dynamics of *TFF1* transcriptional bursts, we utilized an MCF-7 cell line where 3 *TFF1* alleles were endogenously tagged with 24xMS2 stem loops in the 3′ UTR (*TFF1-MS2*).^16^ To control for dose-dependent differences in burst frequency, we determined the saturating concentrations of E2 and each EDC by performing a dose response using a single-molecule fluorescence *in situ* hybridization (smFISH) probe set targeting the exons of *TFF1* (Green, Figures S1A and S1B). MCF-7 cells were hormone depleted for 2 days prior to a 3-day treatment with either E2 or one of the three EDCs, Genistein (Gen), Bisphenol S (BPS), and BPA. Following this treatment, cells were fixed and smFISH was performed. To ensure that the measurements taken using the dose response were accurate, we assayed intermediate concentrations, between 10nM and 100μM, for each of the three EDCs. While 1nM E2 was sufficient to saturate the *TFF1* transcriptional response, 10μM for each EDC was necessary to achieve saturation (Figures 1A and 1B). We extracted half maximal effective concentrations (EC_50_) for each ligand finding the EC_50_ of E2 for *TFF1* was 0.018nM, in agreement with previous studies.^16^^,^^34^^,^^35^ BPA, however, had a substantially higher EC_50_ of 523nM (Table S1). Additionally, for E2 treated cells our dose response plateaus at 114 *TFF1* RNA per cell. Among the EDCs, only BPA failed to induce *TFF1* to the same levels, plateauing at 54 RNA per cell. Thus, each of the tested EDCs required much higher doses to saturate *TFF1* induction and BPA uniquely failed to induce *TFF1* to the same extent as E2, Gen, or BPS. In this study, we use saturating concentrations of E2 (1nM) or EDCs (10μM) for all subsequent assays.

BPA was the only ligand we tested which resulted in a decrease in *TFF1* RNA per cell at saturation. Thus, we treated *TFF1-MS2* cells with E2 or BPA and performed live-cell imaging to determine how BPA treatment affects the bursting dynamics of *TFF1*. When the MS2 stem loops in the 3′-UTR of *TFF1* are transcribed, the loops are bound by several MS2 bacteriophage coat proteins fused to eGFP (Figure S1C). This system allows for visualization of nascent transcription sites (TS) in living cells.^36^ To capture steady state bursting dynamics, we imaged *TFF1*-*MS2* cells that were treated for 8 h (h) with either E2 or BPA (Figure 1C) and tracked individual *TFF1-MS2* TS by extracting their fluorescent intensities.^16^^,^^37^

The bursting dynamics of individual alleles varied in both treatments, with some alleles bursting only once during the recording window, while others burst more frequently (Figure 1D, Videos S1 and S2). We calculated the burst duration and inactive period from 30 fluorescent intensity traces per treatment. Each trace was extracted from randomly sampled alleles across multiple cells. We observed that the distribution of burst duration significantly differed between E2 and BPA treated cells. Additionally, the mean burst duration decreased in cells treated with BPA (burst duration = 13.8min with E2 v 8.43min with BPA *p* < 0.6e-5 Kolmogorov-Smirnov [KS] test, Figure 1E). Conversely, there was no significant difference in the median inactive periods between E2 and BPA treated cells (48.3min v 47.5min respectively, *p* < 0.62, KS test, Figure 1F). These data indicate that BPA can induce maximal *TFF1* burst frequency equivalent to E2, but BPA-induced bursts were shorter than those induced by E2.

Video S1. A TFF1-MS2 cell induced with 1nM E2 for 8h with bursting transcription sites, related to Figure 1 The cell was imaged for 14.2 h. Video is saved at 10 frames per second (fps).

Video S2. A TFF1-MS2 cell induced with 10μM BPA for 8h with bursting transcription sites, related to Figure 1 The cell was imaged for 14.2 h. Video is saved at 10 frames per second (fps).

Next, using our live-cell data, we examined if BPA affected the fraction of cells with transcriptional bursts. This was done by calculating the cumulative fraction of cells that exhibited at least one *TFF1* burst over the course of the time-lapse. We observed 84.6% of cells exhibited at least one *TFF1* burst over the 14.2-h time course in E2 treated cells, while BPA treatment resulted in only 56.7% (Figure 1G). Additionally, we observed a plateau in the fraction of cells with a *TFF1* burst in BPA treated cells after 7.2h of imaging time whereas bursting of *TFF1* in new cells continued for ∼11.4h when treated with E2 (Figure 1G). Thus, BPA treated cells fail to continually activate transcriptional bursts from new alleles. However, our results suggest that the alleles that are transcriptionally active in cells treated with BPA can activate bursts as frequently as alleles in E2 treated cells bursting about once every hour. This data suggests that BPA treatment could affect intrinsic factors regulating transcription of *TFF1* resulting in a smaller fraction of the alleles that are transcriptionally permissive. Alternatively, BPA treatment could have extrinsic effects that alter the availability of transcription factors. However, we cannot distinguish between these with live-cell data alone.

### Fewer *TFF1* alleles are transcriptionally active with BPA treatment

**Figure 2:**
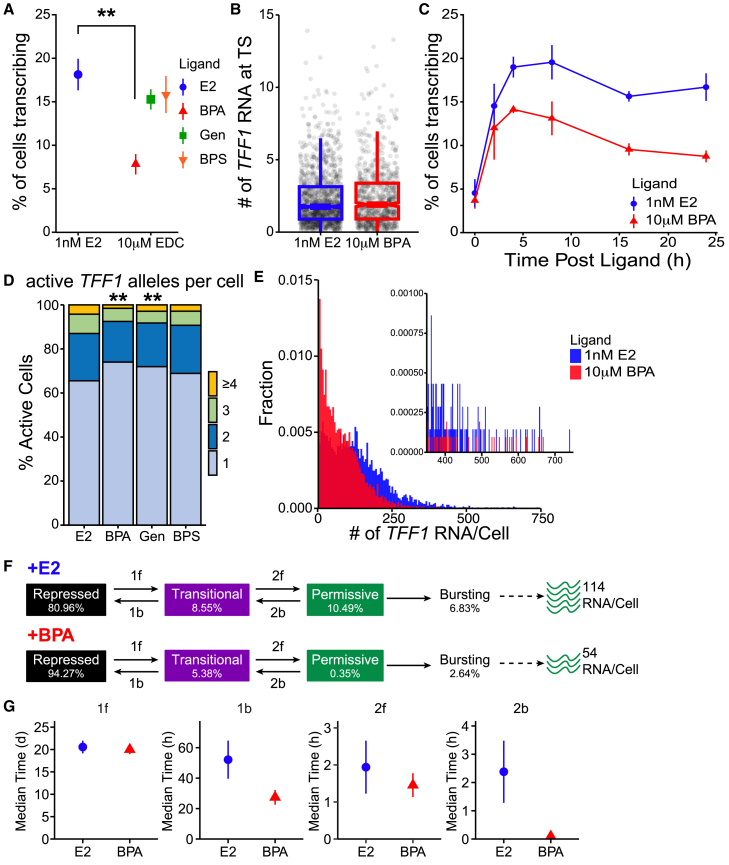
Fewer *TFF1* alleles are transcribed with BPA treatment (A) Average percent of cells transcribing *TFF1* from the dose response data of 3 smFISH replicates represtented by the mean and standard deviation. *p*-values were calculated with t-tests. (B) Boxplot of the number of *TFF1* RNA at TS extracted from smFISH data. *p*-values were calculated with Wilcoxon Rank-Sum Test. (C) smFISH time course showing a time dependent increase in the percent of cells with *TFF1* TS post treatment with ligand. Data shown as the mean and standard deviation of 3 smFISH replicates. (D) Frequency distributions of the number of active *TFF1* TS extracted from the smFISH does response *p*-values were calculated with Wilcoxon Rank-Sum Test. (E) Histogram of the distribution of *TFF1* RNA per cell extracted from smFISH data. (F) Schematic of the mathematical model depicting three regulatory gene states of *TFF1* transcription. Alleles occupy only gene state at a time and occupancy probability reported under each gene state. Fraction of bursting alleles and RNA/Cell extracted from smFISH data. (G) Median duration, extracted from modeling the smFISH and live-cell *TFF1* data, that alleles spend in each state. Error bars represent 95% confidence intervals. ∗*p* ≤ 0.05 ∗∗*p* ≤ 0.01.

Our live-cell imaging data showed that E2 and BPA treatment resulted in similar *TFF1* burst frequency, but burst durations were shorter in BPA treated cells. Moreover, fewer cells contribute to the response. Yet, it is unclear if these differences can account for the more than 50% reduction in *TFF1* RNA we observed from our dose response. Given these observations we extracted transcriptional bursting parameters from our smFISH dose response. In our smFISH images, we defined TS as intron spots (red) that colocalized with exon spots (green) within the nucleus (Figure S1B). Using these criteria, we find that 18.1 ± 1.8% of E2 treated cells had *TFF1* TS while only 7.8 ± 1.2% of BPA treated cells had a TS (Figure 2A). This difference in fraction of cells with TS correlates with a 45% less *TFF1* RNA per cell (Figure S2A). Cells treated with Gen and BPS have a similar fraction of cells with TS as cells treated with E2. Additionally, we observe a dose-dependent increase in the fraction of cells with TS for all ligands (Figure S2B). To test whether the upregulation of *TFF1* expression with BPA treatment is ERα-dependent, we performed smFISH in cells treated with E2 or BPA in the presence of a complete ERα antagonist, Imperial Chemical Industries 182,780 (ICI). A 24-h cotreatment with ICI effectively inhibited the increase in the fraction of cells with a *TFF1* TS observed with E2 or BPA alone (Figure S2C) verifying that BPA activates *TFF1* transcription through ERα consistent with results reported using luciferase assays.^38^

Next, we asked if the 4.29-min reduction in burst duration resulted in less RNA produced at the TS. Generally, shorter *TFF1* transcriptional bursts correlated with fewer RNA output per burst.^16^ We observed a slight increase of 0.3 nascent RNA in the average number of RNA per TS in cells treated with Gen relative to E2 (Welch two-sample t-test, *p* < 0.020). We did not observe changes in cells treated with BPS or BPA (Figure S2D). To ensure that BPA treatment does not result in alterations in the number of RNA produced at each TS we performed an independent replicate. This replicate confirmed our dose response data showing that BPA treatment does not alter the distribution of RNA produced at *TFF1* TS (*p* < 0.387, Wilcoxon rank-sum test, Figure 2B). This indicates that the changes in burst duration extracted from our *TFF1-MS2* data did not result in fewer RNA produced per burst.

We next addressed whether the difference in the number of cells initiating bursts in BPA-treated cells was due to slower kinetics of the *TFF1* transcriptional response. The time course revealed that treatment with both ligands induces transcription at similar rates within the initial 2h of treatment. However, at 4h, there is a difference in the fraction of cells with TS which is maintained up to 24h post treatment (Figure 2C). This result and our data showing no difference in burst frequency between treatments suggests that reduced transcriptional activation by BPA treatment is occurring from either fewer active cells or fewer active alleles. To distinguish between a cellular response or an effect of BPA at the level of single alleles, we extracted the fraction of cells with multiple *TFF1* TS from our smFISH data. We find that the probability of observing cells with more than 1 active TS is reduced in BPA and Gen treated cells compared to cells treated with E2 (*p* < 0.002 and 0.001 respectively, Wilcoxon rank-sum test, Figure 2D). This indicates that BPA is primarily affecting activation of *TFF1* at the single allele level.

Additionally, when the mRNA per cell distributions are plotted, we observe cells treated with BPA explore the same range of RNA per cell as cells treated with E2 (Figures 2E and S2E). Thus, cells treated with BPA, which have multiple active alleles bursting at similar frequencies and amplitudes as those treated with E2, are capable of maximal *TFF1* RNA production. However, due to significantly fewer active alleles in the BPA treated population, the distribution of RNA per cell shifts toward the left, resulting in more cells with little to no *TFF1* RNA expression compared to E2 treated cells. To further quantify these changes, we fit the smFISH RNA distributions simultaneously with the bursting kinetics extracted from the live-cell imaging with a mathematical model applied previously^16^^,^^39^^,^^40^ (Figure S2F). The model consists of transitions between hidden gene states. One of these gene states is the active (permissive) state, where transcription is initiated and the MS2 tagged RNA is transcribed allowing for visualization of the nascent RNA. This data was well fit to a 3-state gene model consisting of a repressed state, transitional state, and permissive state, and 3 reporter states that allowed for 0 to 3 MS2 tagged nascent RNA to be observable simultaneously. The reporter states or bursts can be initiated when the gene is in the permissive state. The model predicted the transition rates, which were used to predict allele occupancy in each transcriptional state and the transition rates between these states. It found that more alleles in BPA treated cells were likely to occupy the repressed state relative to E2 treated cells (94.27% and 80.96% respectively, Figure 2F). The model also predicts that a smaller fraction of alleles in BPA treated cells are in the permissive state (0.35% and 10.49%, respectively). This probability is in line with the fraction of active alleles we observe with smFISH for E2 (6.83%, Figures 2A and 2D). However, given that the tracked MS2 spots are biased to alleles that burst within the observation frame, the model may underestimate the fraction of alleles in BPA treated cells as we observe 2.64% of alleles active by smFISH. Consistent with previous *TFF1* live-cell data,^16^ the model predicts that the transition rate out of the repressed state is very slow, on the order of weeks. Finally, the model predicts that, compared to E2, BPA speeds up the rate at which alleles leave the intermediate state and enter the repressed state, and the rate at which alleles are leaving the permissive state. This is in line with the observation that burst durations were shorter in BPA treated cells (Figure 1F). Other rates were not significantly altered (Figure 2G, Table S2). Taken together, our data show that there are fewer active *TFF1* alleles in cells treated with BPA. However, alleles in both E2 and BPA treated cells are bursting with the similar frequencies, about once every 48 min. We detected shorter *TFF1* bursts in BPA treated cells but our smFISH shows that the same amount of *TFF1* RNA were produced per burst. Our data indicates that Gen and BPS treatments induce *TFF1* bursts with similar kinetics as E2 treated cells and BPA is unique in its effect on the fraction of active alleles.

### BPA treatment results in altered nucleosome positioning at the *TFF1* promoter

**Figure 3:**
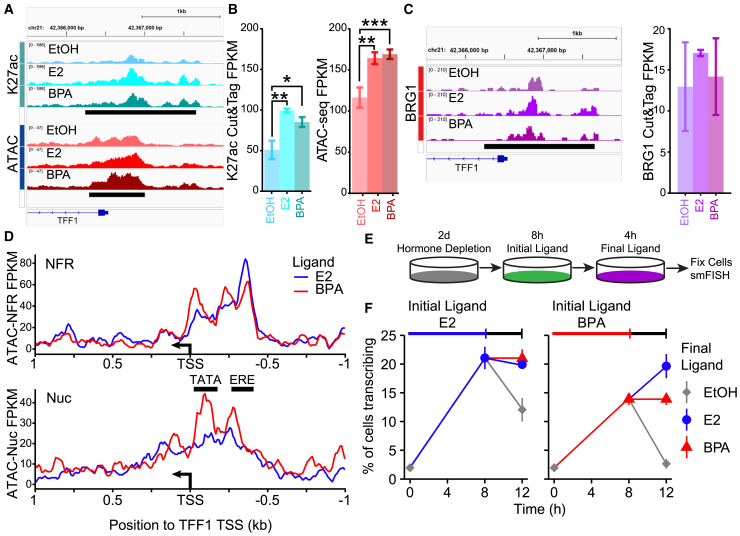
BPA treatment results in increased nucleosome phasing at *TFF1* (A) Browser track of *TFF1* promoter showing K27ac deposition and chromatin accessibility. K27ac and ATAC-seq peaks are annotated by black bars. (B) Quantification of K27ac deposition and accessible chromatin peaks at the *TFF1* promoter. *p*-values determined using DESeq2. Error bars represent the standard deviation of 3 replicates. (C) Browser track of *TFF1* promoter showing BRG1 occupancy and quantification of BRG1 coverage at the K27ac peak. (D) Line plot of the NFR and Nuc coverage extracted at the *TFF1* TSS. Black bars indicated positions of the TATA box nucleosome and ERE nucleosome identified in.^44^^,^^45^ (E) Diagram of the experimental process used in the pretreatment and replacement experiments. (F) Fraction of cells transcribing *TFF1* following washout experiments. Data represented as mean and standard deviation of 3 smFISH repliates. ∗*p* ≤ 0.05 ∗∗*p* ≤ 0.01 ∗∗∗*p* ≤ 0.001.

Upon treatment with BPA, we observed a smaller percentage of cells with *TFF1* TS, and of those cells, fewer had multiple TS. This difference in the fraction of cells with *TFF1* TS shows that cells treated with BPA have fewer alleles in a transcriptionally permissive state. To determine if this correlates with differences at the chromatin level, we performed Cleavage Under Targets and Tagmentation (CUT&Tag) for the active histone modification, H3K27ac (K27ac), and ATAC-seq to measure chromatin accessibility. If fewer alleles are in a transcriptionally permissive state, we would expect to detect fewer reads from the *TFF1* promoter region in both these assays. For our genomic assays, we focused on the 8-h treatment timepoint where we observe the maximum difference in *TFF1* transcription (Figures 1G and 2C). In addition, single-molecule foot printing assays show that TF binding reaches steady state after 8h of induction.^41^ Treatment with either E2 or BPA increased K27ac deposition 1.90 and 1.62-fold respectively (*p* < 0.34e-4 and 0.7e-3, respectively) and chromatin accessibility 1.41 and 1.45-fold respectively (*p* < 0.002 and 0.659e-4 respectively) relative to vehicle control (Figures 3A and 3B). However, contrary to our hypothesis, we did not observe differences between E2 and BPA treatment in either K27ac or accessibility (*p* < 0.19 and 0.8 respectively). This result could corroborate the findings from the mathematical model as the model predicted that alleles in E2 and BPA treated cells exit the repressed state at the same rate. Furthermore, we profiled occupancy of the SWI/SNF ATPase BRG1, which plays a key role in chromatin remodeling through ATP-dependent nucleosome displacement.^42^^,^^43^ BPA treatment does not alter occupancy of BRG1 at the promoter of *TFF1* (Figure 3C) consistent with BPA dependent remodeling of the *TFF1* promoter.

Given that no differences were detected in K27ac or bulk ATAC-seq reads that could account for the reduction in the fraction of cells with *TFF1* TS, we generated additional subsets of our ATAC-seq reads to determine if nucleosome positions were altered by BPA treatment. First, we merged the reads for each condition resulting in >150 million reads per condition and subset the ATAC-seq reads into nucleosome-free regions (NFR, <110bp) and mono-nucleosome length read pairs (Nuc, 150-250bp). In cells treated with E2, the NFR and mono-nucleosome read profiles suggest that the nucleosomes surrounding the transcription start site (TSS) of *TFF1* exhibit broad or “fuzzy” nucleosome positioning (Figures 3D, S3A, and S3B). However, BPA treatment results in NFR and mono-nucleosome read profiles that are more phasic, indicating more consistently positioned nucleosomes across the cell population (Figure 3D). The peaks in the mono-nucleosome read profiles are positioned over the TATA box at −100bp and over an ERE at −330bp, consistent with previously characterized nucleosome positions at *TFF1*.^44^^,^^45^ This structure is disadvantageous for successful transcription initiation because nucleosomes obscure important *cis*-regulatory elements. Additionally, our model suggests that this structure increases the rate at which alleles revert to the repressed gene state. Together, these data suggested that E2-bound ERα produced a transcriptionally favorable nucleosome structure at the *TFF1* promoter whereas BPA-bound ERα could not. As such, BPA-bound ERα appeared to be deficient in the ability to generate a transcriptionally permissive state.

To address if this BPA induced deficiency in chromatin remodeling had long lasting effects on *TFF1* transcription, we performed pretreatment and replacement (washout) experiments. Cells were hormone depleted for 2 days before an initial treatment with either E2, BPA, or vehicle for 8h. Cells were then rinsed with hormone depleted media and treated with the reciprocal compound or vehicle for additional 4h (Figure 3E). As 8h E2 induces the maximal *TFF1* fractional response, an additional 4h of E2 treatment does not further increase the fraction of cells with *TFF1* TS (21.1 ± 1.9% of cells Figure 3F, left panel, blue). Conversely, following 8h of E2 with a 4-h washout with vehicle halved the fraction of cells with *TFF1* TS, indicating that maintenance of *TFF1* transcription was ligand-dependent (gray). Following E2 treatment with BPA treatment resulted in maintenance of the fraction of cells with *TFF1* TS (21.0 ± 1.6% of cells, Figure 3F, left panel, red). These data suggest that BPA bound ERα can activate alleles prepatterned by E2 treatment without any impediment.

As with our time course, 8h of BPA treatment induces a *TFF1* response that is lower than that induced by E2 (Figure 3F, right panel, red). Following this with an additional 4h of BPA maintains the response. Washing out the initial BPA treatment with vehicle reduces the fraction of cells with *TFF1* TS to baseline (Figure 3F, right panel, gray), indicating that BPA does not induce persistent *TFF1* transcription. Finally, following BPA with 4h E2 increased the fraction of cells with *TFF1* TS to the maximal level observed in E2 treatment (19.6 ± 2.1% of cells Figure 3F, right panel, blue) which is comparable to the E2 alone treatment. This indicates that E2-bound ERα was able to activate transcription at alleles that BPA-bound ERα could not. Together, these results suggest that nucleosome positioning at the promoter of *TFF1* is not static, and that E2 bound ERα with its associated cofactors can efficiently remodel the locus. This remodeling persists upon E2 removal, or challenge with BPA.

### Nucleosome phasing is a hallmark of genes with reduced fractional responses to BPA

**Figure 4:**
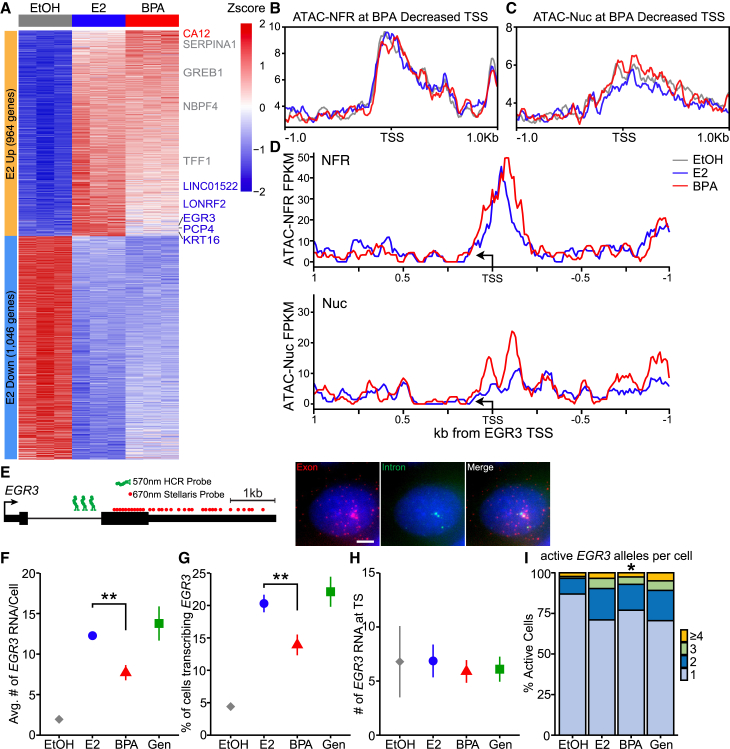
Nucleosome phasing correlates with reduced fraction responses (A) Heatmap of RNA-seq results for E2 responsive DEGs. Gene symbols indicate genes up regulated (red) and down regulated (blue) over E2 by BPA. (B) Meta profile of the NFR signal at the TSS of the top BPA down regulated genes. (C) Meta profile of the Nuc signal at the TSS of the top BPA down regulated genes. (D) Line plot of the NFR and Nuc signal extracted at the *EGR3* TSS. (E) Schematic of the *EGR3* transcript and approximate positions of RNA smFISH and intron HCR probes. Representative image of *EGR3* smFISH from MCF-7 cells, RNA visible as red spots, TS are visible where RNA and intron spots colocalize. Scale bar represents 5μm. (F) smFISH results of the average *EGR3* RNA accumulation in cells treated with E2 or BPA. The mean and standard deviation of 3 replicates is represented. *p*-values were calculated with t-tests. (G) smFISH results of the average fraction of cells transcribing *EGR3* in cells treated with E2 or BPA. Mean and standard deviation calculated from 3 replicates. (H) Average number of nascent *EGR3* RNA at active TS. Data represented as the mean and standard deviation of 3 smFISH replicates. (I) Frequency distributions of the number of active *EGR3* TS per cell *p*-values were calculated with Wilcoxon Rank-Sum Test. ∗*p* ≤ 0.05 ∗∗*p* ≤ 0.01.

To further investigate the effects of BPA treatment on other late E2 responsive genes, like *TFF1*, we performed RNA-seq after 2 days of hormone depletion cells were treated with E2 or BPA for 24h. We called differentially expressed genes (DEGs) using a false discovery rate (FDR) of less than 0.05 and an absolute fold change greater than 1.5. We identified 964 upregulated and 1,046 downregulated DEGs in E2 treated cells (Figures 4A and S4A). Similarly, BPA treated cells had a robust response with 831 upregulated and 837 downregulated DEGs (Figure S4B). The majority, 70%, of E2 target genes were also induced by BPA, including *TFF1*, which was significantly upregulated in both datasets (*p* < 0.218e-268 and 0.387e-265, respectively). We next asked how many genes were differentially expressed between E2 and BPA treatments. Keeping our FDR cutoff at 0.05, we identified 376 upregulated and 331 downregulated DEGs with more than a 10% difference between the treatments (Figure S4C). When we considered only E2 DEGs, we found that 14% of this gene set had a significantly altered responses with BPA treatment (Figures 4A and S4D). *TFF1* was not among this set of BPA altered E2 DEGs, failing to pass our FDR threshold. However, smFISH experiments demonstrate that BPA treatment reduces *TFF1* mRNA accumulation (Figure S2A).

Next, we selected the 54 E2 upregulated genes that were reduced by greater than 30% with BPA treatment. Using publicly available short 5′-capped RNA sequencing data, we identified the predominant TSS that is expressed in MCF-7 for each gene^46^ and examined the chromatin accessibility over these sites. The ATAC NFR signal is similar between treatments. However, there is a slight depletion at the TSSs in BPA treated cells, making them more similar to vehicle treated cells than to E2 treated cells (Figure 4B). The mono-nucleosome signal shows increased levels surrounding these TSSs and increased nucleosome phasing in BPA treated cells. This pattern suggests that the deficient nucleosome remodeling observed at *TFF1* in BPA-treated cells may represent a more general phenomenon (Figure 4C). BPA-induced nucleosome phasing at the TSS is particularly prominent at *EGR3* (Figure 4D). In addition to this phasing at the TSS of *EGR3* there is similar phasing under the ERα peak identified by ChIP-seq approximately 2kb up stream of the TSS^47^ (Figure S4E). Our RNA-seq shows *EGR3* expression is decreased by 30.7% in BPA treated cells relative to E2 treatment. Thus, we identified *EGR3* as a candidate to determine if treatment with BPA results in a smaller fraction of active alleles. To test this, we designed smFISH probes to the intron and last exon of *EGR3* (Figure 4E). First, we confirmed that 3 days of BPA treatment results in a reduction of *EGR3* RNA per cell compared to E2 treatment (*p* < 0.005, Figure 4F). Similar to *TFF1*, BPA treatment resulted in a corresponding decrease in the fraction of cells with *EGR3* TS and did not alter the number of nascent *EGR3* transcripts (Figures 4G and 4H). Finally, with BPA treatment, the probability of observing multiple *EGR3* TSs per cell is decreased, relative to E2 (Figure 4I). Thus, BPA-bound ERα appears to be deficient in its ability to induce the appropriate chromatin remodeling, at E2-induced genes that are not fully activated by BPA treatment. This leads to nucleosome phasing and a lower fraction of active TS for both genes, suggesting that this remodeling activity is necessary for full induction of transcriptional activity.

### ERα recruitment to *TFF1* and *GREB1* promoters reflects differential transcriptional responses to BPA

**Figure 5:**
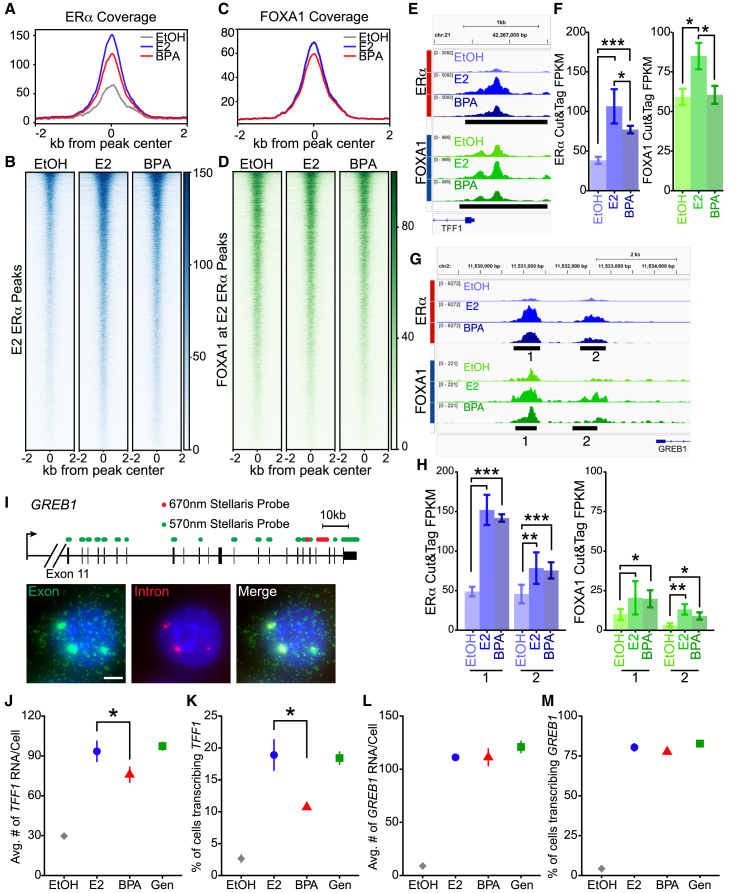
BPA mediated effects on ERα recruitment are gene specific and correlate to the fractional response (A) Meta profile of ERα occupancy at 12,223 peaks identified after 8h of E2 treatment. *p*-values were calculated by using the average signal of a 500 bp window centered on the peak center and a Wilcoxon Rank-Sum test was performed. (B) Heatmap of ERα occupancy at E2-ERα peaks. (C) Meta profile of FOXA1 occupancy at E2-ERα peaks. (D) Heatmap of FOXA1 occupancy at E2 ERα peaks. (E) Browser track of the ERα and FOXA1 peaks at the *TFF1* promoter. Peaks are annotated by black bars. (F) Quantification of ERα and FOXA1 peaks at the *TFF1* promoter. *p*-values extracted using DESeq2. Error bars represent standard deviation. (G) Browser track of the ERα and FOXA1 peaks at the *TFF1* promoter. Peaks are annotated by black bars and denoted by 1 or 2. (H) Quantification of ERα and FOXA1 peaks at the *GREB1* promoter. *p*-values extracted using DESeq2. Error bars represent standard deviation. (I) Schematic of the *GREB1* transcript and approximate positions of RNA and intron smFISH probes. Representative image of *GREB1* smFISH from MCF-7 cells, RNA visible as green spots, transcriptional bursts are visible where RNA and intron spots colocalize. Scale bar represents 5μm. (J) smFISH results of the average *TFF1* RNA accumulation in cells treated with vehicle (EtOH), E2, BPA or Gen. The mean and standard deviation of 3 replicates is represented. *p*-values were calculated with t-tests. (K) smFISH results of the average fraction of cells transcribing *TFF1* in cells treated with EtOH, E2, BPA or Gen. Mean and standard deviation calculated from 3 replicates. *p*-values calculated with t-tests. (L) smFISH results of the average *GREB1* RNA accumulation in cells treated with EtOH, E2, BPA or Gen. The mean and standard deviation of 3 replicates is represented. *p*-values calculated with t-tests. (M) smFISH results of the average fraction of cells transcribing *GREB1* in cells treated with EtOH, E2, BPA or Gen. Mean and standard deviation calculated from 3 replicates. *p*-values calculated with t-tests. ∗*p* ≤ 0.05 ∗∗*p* ≤ 0.01 ∗∗∗*p* ≤ 0.001.

To investigate whether this enhanced nucleosome phasing at ERα binding sites altered recruitment, we performed CUT&Tag of ERα and FOXA1. FOXA1 is a pioneering factor that commonly co-binds at ERα binding regions and is one of the main drivers in the establishment of ERα-mediated transcriptome in response to E2.^21^^,^^48^^,^^49^ We identified a total of 16,059 ERα peaks with samples clustering by treatments (Figures S5A and S5B). In cells treated with E2, 12,223 peaks were identified. The majority of these peaks, 73% (8,932 peaks), overlap with peaks identified in previously published ERα ChIP-seq data (Figure S5C). Approximately two-thirds of the peaks identified in BPA treated cells overlapped with peaks identified in E2 treated cells (Figure S5A). We focused the rest of our analysis on the 12,223 ERα peaks identified in cells treated with E2 only (Figures 5A and 5B). We observe that BPA treatment leads to ERα recruitment at the majority of the E2 peaks however, ERα occupancy was significantly reduced relative to E2 treatment (*p* < 0.138e-26, Figures 5A and 5B). This reduction in ERα recruitment correlated with a 12.7% reduction in transcriptional response of E2 DEGs (Figure S5D). We next extracted FOXA1 signal at the same ERα peaks (Figures 5C and 5D). BPA treatment resulted in a modest, but significant reduction in FOXA1 recruitment to the set of peaks neighboring ERα bound regions (*p* < 0.0436, Figure 5C). The observed decrease in ERα and FOXA1 recruitment is not due to altered cellular levels of either protein in BPA treated cells compared to E2 (Figure S5E).

The promoter proximal ERα peak at *TFF1* falls directly over the phased nucleosome in BPA treated cells containing the ERE (Figure 3D) and is significantly reduced by more than 25% in the presence of BPA. This reduction in ERα recruitment was mirrored by a decrease in FOXA1 recruitment (Figures 5E and 5F). Thus, altered nucleosome phasing was concomitant with reduced ERα and FOXA1 binding at the *TFF1* promoter. However, not all ERα peaks are reduced with BPA treatment. The ERα and FOXA1 peaks at the promoter of the predominant *GREB1* transcript were not altered by BPA (Figures 5G and 5H). *GREB1* is another well characterized E2 responsive gene that was highly up regulated with E2 treatment in our RNA-seq (Figure 4A). *TFF1* and *GREB1* are intriguing candidates to determine if ERα and FOXA1 recruitment to promoters reflected the fraction of cells with TS. We designed probes for *GREB1* and performed smFISH (Figure 5I). As we showed in our dose response (Figures 1A and S2A), BPA induction significantly reduced *TFF1* RNA accumulation by reducing the fraction of cells with *TFF1* TS (Figures 5J and 5K, *p* < 0.045 and 0.029 respectively). However, BPA does not alter *GREB1* RNA accumulation or the fraction of cells with *GREB1* TS (Figures 5L and 5M). Treatment with Gen resulted in a similar levels of RNA accumulation and fraction of transcribing cells for both *TFF1* and *GREB1*, suggesting that the change in bursting is specific to BPA (Figures 5J–5M). We do not observe a differential effect of ligands, relative to E2 treatment on the number of nascent transcripts produced by both genes (Figure S5F). We confirm that the probability of observing multiple *TFF1* TS in BPA treated cells is decreased. However, unlike *TFF1,* BPA not only has no effect the fraction of cells with *GREB1* TS but does not reduce the probability of observing multiple TS. On the other hand, Gen treatment moderately increases the probability of observing multiple *GREB1* TS (Figure S5G). These results indicate that ligand-bound ERα and FOXA1 recruitment to the promoters of *TFF1* and *GREB1* correlate with the fraction of cells transcribing those estrogen responsive genes.

To bolster this finding, we further integrated our ERα CUT&Tag and RNA-seq data. We identified two transcripts, *LINC01522* and *LONRF2*, which exhibit similar behavior as *TFF1*. Specifically, ERα occupancy at peaks associated with these transcripts diminish following BPA treatment, corresponding with a decrease in their transcript levels (Figure S6A). Additionally, BPA does not alter the RNA levels or ERα peaks associated with *SERPINA1* and *NBPF4* (Figure S6A). Interestingly there was a small group of E2 upregulated DEGs that were significantly increased by BPA treatment. Specifically, *CA12* exhibited increased ERα occupancy at an associated ERα peak with BPA treatment, indicating that more ERα binding can result in higher RNA levels (Figure S6A). Finally, to confirm that our results were not phenomena unique to MCF-7 cells, we performed smFISH in T-47D cells. Since *TFF1* expression in T-47D cells is negligible, we focused our analysis on the ER responsive targets *EGR3* and *GREB1*. We observed increases in the fraction of cells with *EGR3* TS after 24h of E2 or BPA treatment. Similar to the results observed in MCF-7 cells, BPA fails to induce *EGR3* transcription to the same level as E2 (Figure S6B, *p* < 0.014). In T-47D cells and there is no effect of BPA on *GREB1* transcription (Figure S6C, *p* < 0.068). These data uncovered gene and ligand specific regulation of transcriptional bursting in ERα signaling. Furthermore, the fractional response to ligands is conserved across ERα-positive breast cancer cell lines.

### BPA bound ERα disrupts enhancer-promoter synergy at the *TFF1* locus through cofactor recruitment

**Figure 6:**
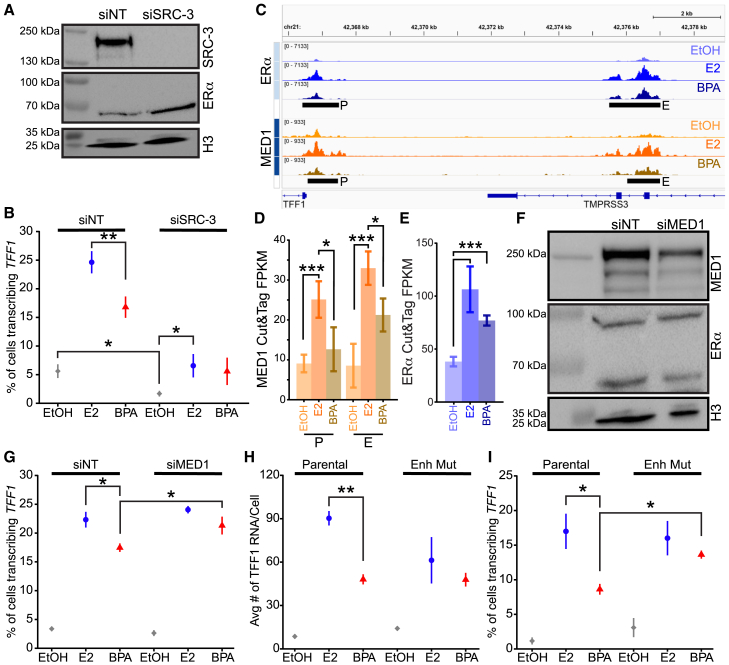
BPA-bound ERα impairs enhancer mediated *TFF1* transcription (A) WB of nuclear protein fraction using SRC-3 (100% knockdown), ERα or H3 specific antibodies from cells transfected with non-targeting (siNT), SRC-3 (siSRC-3). (B) The average fraction of cells transcribing *TFF1* from 3 replicates in MCF-7 cells transfected with siNT or siSRC-3 siRNA pools. Error bars represent standard deviation, *p*-values calculated with t-tests. (C) Browser tracks of ERα and MED1 treated with either EtOH, E2 or BPA for 8h. *TFF1* promoter (P) and enhancer (E) indicated by black bars. (D) Quantification of MED1 at the promoter and enhancer peaks of *TFF1*. *p*-values determined using DESeq2. Error bars represent standard deviation. (E) Quantification of ERα at the *TFF1* enhancer peak, error bars represent standard deviation. *p*-values determined using DESeq2. (F) WB of nuclear protein fraction using MED1 (bands from top to bottom: 52.4%, 54.5%, and 60% knockdown) ERα or H3 specific antibodies from cells transfected with non-targeting (siNT), MED1 (siMED1). A nonspecific band at 100 kDa is present in the WB probed for ERα and is the result of suboptimal stripping of the MED1 antibody. (G) The average fraction of cells transcribing *TFF1* from 3 replicates in cells transfected with siNT or siMED1 siRNA pools. Error bars represnt standard deviation. (H) smFISH results of the average *TFF1* RNA accumulation in E2 or BPA treated parental or *TFF1-MS2* enhancer mutant cells. Error bars represent standard deviation. (I) The average fraction of cells transcribing *TFF1* in parental or *TFF1* enhancer mutant cells. Data plotted as means ± SD. *p*-values calculated with t-tests. ∗*p* ≤ 0.05 ∗∗*p* ≤ 0.01 ∗∗∗*p* ≤ 0.001.

Our data shows that treatment with BPA results in increased nucleosome phasing at the promoter of *TFF1* and a reduction in recruitment of ERα and a pioneer factor FOXA1. This correlates with fewer *TFF1* TS in BPA treated cells. However, BPS treatment results in a similar fraction of cells with *TFF1* TS relative to E2 (Figure 2A). Microarray Assay for Real-Time Coregulator-Nuclear receptor Interactions has shown that there are peptides from 8 proteins that interact with ERα bound by both E2 and BPS, but not BPA.^31^ We identified two of these proteins, SRC-3 and MED1, which are important cofactors for ERα function in response to E2.^26^^,^^27^ We further investigated the impact of depleting these two proteins on *TFF1* transcriptional bursts using siRNA knockdowns (siRNA KD) followed by smFISH. We employed validated SRC-3 (siSRC-3), MED1 (siMED1) specific or non-targeting (siNT) siRNA pools to reduce SRC-3 or MED1 levels. One day after transfection, the cells were treated with either E2, BPA, or vehicle control for 24h. We observed a near total knockdown of SRC-3 protein (Figure 6A). In line with our previous results (Figures 2B; 5K), smFISH of control cells transfected with siNT showed the expected significant decrease in the fraction of cells with *TFF1* TS in BPA treated cells compared to E2 (Figure 6B). This demonstrated that transfection with the non-targeting siRNA pools did not alter the BPA response. In contrast, siSRC-3 transfection results in a significant decrease in the fraction of cells with *TFF1* TS in both E2 and BPA treatments (*p* < 0.379e-3 and 0.371e-3 respectively). In addition, we observed a significant reduction in the fraction of vehicle treated cells with *TFF1* TS in siSRC3 transfected cells compared to the respective siNT control (*p* < 0.018) indicating that SRC-3 has a role in the baseline expression of *TFF1*. Additionally, the difference in the relative fraction of cells with *TFF1* TS for the E2 and BPA treatments is lost after SRC-3 knockdown (*p* < 0.618, Figure 6B). Taken together, these findings indicate that SRC-3 has a role upstream of ERα in both E2 and BPA mediated activation of *TFF1*. Moreover, our data indicates that SRC-3 is crucial for determining the fraction of alleles that can initiate *TFF1* bursts in response to E2.

The Mediator complex is important for regulating promoter and enhancer interactions. The primary enhancer of *TFF1* is located <10kb upstream of the gene. This enhancer plays a critical role in activation of *TFF1* through recruitment of transcription factors and cofactors including ERα and FOXA1.^22^^,^^47^^,^^50^^,^^51^ Our CUT&Tag data agrees with these findings in that we observe ERα and FOXA1 recruitment to the enhancer region, as well as deposition of K27ac at this enhancer which is highly enriched with E2 treatment (Figures S7A and S7B). We tested if MED1 recruitment was perturbed in BPA treated cells by profiling MED1 occupancy at the *TFF1* locus with CUT&Tag (Figure 6C). We found that MED1 is recruited to both the *TFF1* promoter and enhancer (Figure 6D). Recruitment to the promoter and enhancer is significantly reduced with BPA treatment (*p* < 0.003 and *p* < 0.049, respectively, Figure 6D). As we previously showed, ERα recruitment is reduced at the *TFF1* promoter (Figure 5F). However, we did not observe a significant difference in ERα recruitment between E2 and BPA at the enhancer (*p* < 0.056 Figure 6E). This data suggests that BPA treatment perturbs the *TFF1* locus as a whole, specifically affecting ERα and MED1 occupancy.

Next, we assayed transcriptional bursting of *TFF1* following siRNA targeting MED1. Following siMED1 transfection we observe at least 50% knockdown of MED1 protein (Figure 6F). Knockdown of MED1 in cells treated with E2 did not elicit any change in the fraction of cells with *TFF1* TS (Figure 6G). Interestingly, MED1 knockdown in BPA treated cells prevented the BPA-mediated reduction in transcriptional response of *TFF1* (*p* < 0.075, Figure 6G). In this regard, it is possible that the reduction in MED1 levels may allow for substitution or compensation by other mediator subunits, potentially forming a more effective initiation complex with BPA-bound ERα to create a functional complex. Together our results suggest that BPA treatment leads to the formation of a dysfunctional ERα-mediated protein complex at the *TFF1* enhancer.

To determine the extent of the enhancer dysfunction in BPA treatment, we used a previously described MCF-7 cell line where the proximal enhancer region was partially deleted (*TFF1* enhancer mutant).^16^ This deletion removes several transcription factor binding sites including FOXA1, while leaving the major ERα binding site intact (Figure S7C). Following treatment with E2, BPA, or vehicle, we quantified RNA accumulation per cell and the fraction of cells with *TFF1* TS using smFISH. As expected in the *TFF1* enhancer mutant cells, *TFF1* RNA accumulation with E2 treatment, is reduced by 32%. This data confirms the importance of the enhancer in activation of *TFF1*. However, the difference in *TFF1* RNA accumulation between E2 and BPA treatment is lost in *TFF1* enhancer mutant cells (Figure 6H). Additionally, in line with our siMED1 results, BPA or E2 treatment of *TFF1* enhancer mutant cells both result in similar fraction of cells with TS (Figure 6I). These cells also exhibit a similar fraction of cells with TS as parental cells. This discrepancy between fraction of cells with bursts and RNA counts can be explained by a previous live-cell study of this enhancer deletion. Lower RNA counts per cell is the result of a 1.9-fold increase in the length of inactive periods between bursts.^16^ In summary, our findings suggest that BPA disrupts the synergy between the *TFF1* promoter and enhancer. This enhancer dysfunction limits the ability of the BPA-ERα complex to increase the number of *TFF1* alleles that can initiate transcriptional bursts.

## Discussion

**Figure 7:**
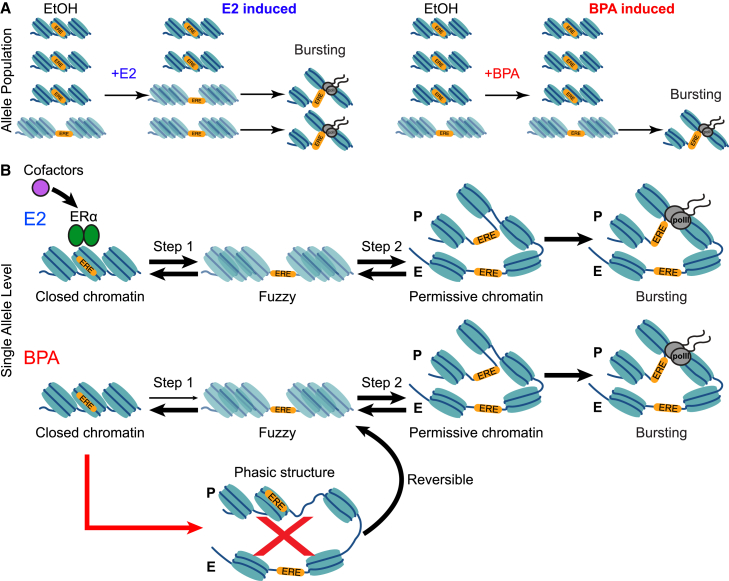
Transcriptional state transitions mediated by ERα (A) population level model of individual alleles treated with E2 and BPA. (B) Model of state transitions mediated by ERα. E2-bound ERα can facilitate step 1 and 2 transitions while BPA-bound ERα primarily facilitates step 2 transitions. BPA-bound ERα results in altered chromatin structure that does not result in a transcriptionally permissive state.

While activation of estrogen responsive genes by EDCs has been known for decades, it remains unknown how EDCs affect the mechanisms of transcriptional bursting. We employed a combination of single-molecule imaging and genomics approaches to gain mechanistic insights into transcription meditated by ERα and its cofactors when bound by. This approach allowed us to build a model of how BPA bound ERα regulates discrete steps in the transcription of specific estrogen responsive genes (Figure 7). We find that BPA treatment can maximally activate specific alleles, inducing bursting with similar frequencies and producing similar numbers of nascent RNAs as in E2 treated cells. This is surprising in light of prior literature indicating that many genes are regulated through frequency modulation.^3^^,^^15^^,^^16^^,^^40^^,^^52^^,^^53^^,^^54^ However, our data show that BPA treatment appears to primarily activate alleles that are already in a transcriptionally permissive state. Whereas ERα bound by E2 can transition new alleles to a permissive state, thereby increasing the pool of active alleles. SRC-3 appears to be critical for maintaining this pool of permissive alleles in hormone depleted conditions. Additionally, BPA bound ERα lacks the cofactors necessary to transition permissive alleles (Figure 7A). At the single allele level our data suggests that *TFF1* alleles are cycling between transcriptional states and ERα has a dual function in this cycling. ERα can activate alleles that are permissive as well as bring new alleles to the transitional state. Modeling of our single-molecule imaging data and our ATAC-seq corroborates this framework. Our modeling suggests the alleles in BPA treated cells can move from the repressed state to the transitional state at the same rate, approximately every 20 days, as E2 treated cells. Our bulk ATAC-seq reads show no difference in accessibility at the promoter of *TFF1*, suggesting an open chromatin structure may mark the transitional state. However, further analysis of the ATAC-seq shows that BPA results in a phasic structure at the *TFF1* TSS. Additionally, our modeling shows that *TFF1* alleles move from the transitional state to the repressed state twice as fast in BPA treated cells relative to cells treated with E2. Taken together this suggests that the phasic structure surrounding the TSS of *TFF1* is not conducive to transcriptional activation and leads to repression of *TFF1* alleles (Figure 7B). However, our modeling also shows that the transition between the transitional and permissive state is unaffected by BPA treatment. This is in line with a model where alleles which have a fuzzy nucleosome structure in hormone depleted conditions can be activated at the same rate by BPA bound ERα as E2 bound ERα. We show that similar to *TFF1*, there are additional 54 E2 up-regulated genes that have increased nucleosome phasing at the TSS with BPA treatment. *EGR3* is among these genes and single-molecule imaging shows that the fraction of active alleles is reduced with BPA treatment suggesting that a similar mechanism we describe for *TFF1* regulates *EGR3* transcription. Our proposed model (Figure 7) links chromatin states to the fraction of active alleles, explaining the lowered RNA output of ERα target genes with BPA treatment.

We observed global reductions in ERα occupancy with BPA treatment. This is in line with previous findings in T-47D cells where BPA treatment reduces ERα occupancy genome wide.^33^ However, in this prior study ChIP was performed following treatment with 100nM BPA, which is well below the EC_50_ of 523nM for BPA observed here. Thus, our findings highlight that even at saturating concentrations of BPA, ERα occupancy is affected. It remains a possibility that structural changes in the LBD of ERα, observed with X-ray crystallography,^29^ propagate to the DNA binding domain altering the interactions of ERα with DNA. However, direct evidence is scant. Molecular Dynamics (MD) simulations of full-length ERα suggest that BPA bound ERα may have perturbed DNA recognition.^55^ Yet, previous ChIP studies^33^ and our CUT&Tag suggest that the majority of ERα peaks, while reduced, with BPA treatment are still occupied.

Additionally, our work highlights the cooperativity between a pioneer factor, FOXA1 and the complex recruited by ERα. While cooperativity between FOXA1 and a non-pioneer factor, HNF4A, has been shown in lymphoblast cells,^56^ the results presented here may be the first showing the ligand dependency of FOXA1 cooperativity with other factors. In addition to the reduction in FOXA1 occupancy at the promoter of *TFF1,* we also observed a reduction in MED1. Previous work using bulk cell data showed that MED1 helps initiate the first phase of transcription of E2-responsive genes within 20 min after E2 stimulation. In the second phase, SRCs recruits p300 to ERα which results in a sustained estrogen regulated transcriptional response.^57^ Here, we propose that the sustained *TFF1* response is initiating from individual alleles transitioning into a permissive state. Additionally, the single-cell data generated in our siSRC-3 KD shows SRC-3 is critical for maintaining *TFF1* alleles in transcriptionally permissive states even in hormone depleted cells. Finally, our work suggests that there are gene specific requirements for transitioning alleles to permissive transcriptional states. For example, while both *TFF1* and *EGR3* were targets for BPA-dependent nucleosome phasing and a reduced fraction of cells with TS, *GREB1* is unaffected by BPA treatment. In this regard, it has been shown that distinct subsets of cofactors are necessary for maximal activation of different E2 responsive genes.^58^

Our work elucidates how the magnitude of the estrogen response is regulated at the single-cell level by the fraction of transcriptionally permissive alleles. ERα and the cofactors it interacts with are critical to establishing the permissive state of the underlying chromatin. EDCs in general, and BPA specifically, are known to disrupt these cofactor interactions with ERα. Here, we demonstrate that BPA bound ERα results in altered chromatin remodeling and fewer permissive alleles. Our data underscore the critical role of epigenetic priming of the chromatin architecture in individual alleles prior to BPA exposure. Our results demonstrate that EDCs and specifically BPA can activate primed alleles, suggesting that BPA’s impact may vary depending on estrogen status of an individual. The EDCs could drive the ectopic expression of estrogen responsive genes from developmental through menopause, where the pre-established chromatin configuration would dictate the extent of transcriptional disruption, making age a significant factor in how BPA influences estrogen-responsive genes. Finally, our observation provides the cornerstone for understanding the transcriptional response mediated by the nuclear receptors. Proper cofactor recruitment is necessary to generate more permissive alleles, and the fraction of these permissive alleles determines the extent of the response of a specific gene. The nuclear receptor itself is key to mediating these interactions at a specific locus. Thus, we speculate that the context of cofactors expressed in specific tissues is a key determinant of the fractional cell response for estrogen responsive genes. Future research will focus on how recruitment and levels of cofactors guide promoter structural rearrangements which are critical for transcriptional bursting.

### Limitations of the study

We note several caveats to the results and methodology presented here, specifically our live-cell imaging approach. This is both a technical and biological caveat. First, we are limited by the biological constraints of the system. The MCF-7 cells move and divide during the imaging window. We thus are constrained to tracking alleles in cells that remain within the field of view and do not divide during the imaging window. Once a cell divides, we lose certainty in our ability to track the same allele. This sets a theoretical max imaging time at 30h or approximately one cell cycle. Additionally, photobleaching and phototoxicity are constraints of long timescale imaging, especially when attempting to maintain high spatiotemporal resolution. Thus, the system only allows for direct observations of long inactive periods that are limited to about 14h, or the length of imaging. However, our modeling suggests these periods for *TFF1* alleles are much longer and advances in imaging methodology would need to be made to directly observe them.

## Resource availability

### Lead contact

The lead contact is Joseph Rodriguez. Please address correspondence to Joseph.Rodriguez@nih.gov.

### Materials availability

Materials generated for or used within this study are available upon correspondence.

### Data and code availability

• The raw data and processed data files are for next generation sequencing experiments are available through GEO Series accession numbers GSE251654.
• This paper does not report original code.
• Any additional information required to reanalyze the data reported in this paper is available from the lead contact upon request.

## Acknowledgments

We would like to thank the Fluorescence Microscopy and Imaging Center (10.13039/100000066NIEHS), specifically Jeff Tucker and Erica Scappini, for use of the Andor Dragonfly Spinning Disk Confocal/HILO Microscope. We also thank the Epigenomics and DNA Sequencing Core (NIEHS) Jason Malphurs and Brian Papas for sequencing and initial QC of CUT&Tag/ATAC-seq libraries. We thank Krystal Orlando and Yang Liu (NIEHS/10.13039/100000002NIH) for trouble shooting CUT&Tag/ATAC-seq with us; Jackson Hoffman (NIEHS/NIH) for his support and encouragement on this project; Krystal Orlando, Harriet Kinyamu, and Paul Wade (NIEHS/NIH) for feedback. Jackson Hoffman (NIEHS/NIH), Richard N. Day (IUPUI), and Murali Palangat (10.13039/100000054NCI/NIH) for critical reading of the manuscript. This research was supported by the National Institutes of Health, 10.13039/100000066National Institute of Environmental Health Sciences ES103331 to J.R. Research was supported by the Intramural Research Program of the NIH, 10.13039/100000062NIDDK to C.C.C. This work utilized the computational resources of the NIH HPC Biowulf cluster (https://hpc.nih.gov).

## Author contributions

Data acquisition: C.R.D., P.Y., and G.A., Data analysis: C.R.D., P.Y., B.D.B., C.C.C., and J.R.; Manuscript preparation: C.R.D., P.Y., B.D.B., C.C.C., and J.R.

## Declaration of interests

The authors declare no competing interests.

## STAR★Methods

### Key resources table

**Table:** 

REAGENT or RESOURCE	SOURCE	IDENTIFIER
**Antibodies**		

ERα	Santa Cruz	Cat# sc-8005; RRID: AB_627556
H3K27ac	Invitrogen	Cat# MA5-23516; RRID: AB_2608307
FOXA1/HNF3α	Cell Signaling Technology	Cat# 53528; RRID:AB_2799438
MED1	Cell Signaling Technology	Cat# 51613; RRID: AB_2799397
SRC-3	Cell Signaling Technology	Cat# 21265
BRG1	Novus Biologicals	Cat# NB100-2594; RRID: AB_2191852
Rabbit (DA1E) mAb IgG XP Isotype Control	Cell Signal Technology	Cat# 3900; RRID: AB_1550038
Mouse (G3A1) mAb IgG1 Isotype Control	Cell Signal Technology	Cat# 5415; RRID: AB_10829607
Rabbit Anti-Mouse IgG H&L	Abcam	Cat# ab46540; RRID: AB_2614925
Guinea Pig anti-rabbit secondary	Antibodies Online	Cat# ABIN101961; RRID: AB_10775589
Beta actin	Proteintech	Cat# 66009-1-Ig; RRID: AB_2687938
Histone 3	Proteintech	Cat# 17168-1-AP; RRID: AB_2716755
Mouse anti-rabbit IgG-HRP	Santa Cruz	Cat# sc-2357; RRID: AB_628497
Goat anti-mouse IgG-HRP	Santa Cruz	Cat# sc-2005; RRID: AB_631736

**Chemicals, peptides, and recombinant proteins**		

17-β-estradiol (E2)	Sigma-Aldrich	Cat# E2758
Bisphenol-A (BPA)	Sigma-Aldrich	Cat# 239658
Bisphenol-S (BPS)	Sigma-Aldrich	Cat# 43034
Genistein (Gen)	Sigma-Aldrich	Cat# G6649
Imperial Chemical Industries 182,780 (ICI)	Sigma-Aldrich	Cat# 5310420001
Formamide (99.5%)	ThermoFisher Scientific	Cat# BP228-100
20x SSC	Quality Biological	Cat# 351-003-131
TWEEN 20	Sigma-Aldrich	Cat# P7949
Heparin sodium salt from porcine intestinal mucosa	Sigma-Aldrich	Cat# H3149
Denhardt’s Solution 50x	Sigma-Aldrich	Cat# D2532
Dextran sulfate sodium salt from Leuconostoc spp.	Sigma-Aldrich	Cat# D8906
ProLong Gold Antifade Mountant with DAPI	ThermoFisher Scientific	Cat# P36935
18 mm #1.5 Coverslip	Electron Microscopy Sciences	Cat# 72222-01
MEM	ThermoFisher Scientific	Cat# 10370
Phenol Red-Free MEM	ThermoFisher Scientific	Cat# 51200
Penicillin/Streptomycin	Sigma-Aldrich	Cat# P0781
L-Glutamine (200 mM)	ThermoFisher Scientific	Cat# 25030081
FBS	Sigma-Aldrich	Cat# F2442
Charcoal/Dextran Treated FBS	Sigma-Aldrich	Cat# F6765
Protein AG-Tn5	Epicypher	Cat# 15-1117
BioMag Plus Concanavalin A (3mL)	Bangs Laboratories	Cat# BP531
Digitonin	Sigma-Aldrich	Cat# 300410
Spermidine	Sigma-Aldrich	Cat# S0266-1G
SYBR Green I Nucleic Acid Gel Stain, 10,000X	ThermoFisher Scientific	Cat# S7563

**Critical commercial assays**		

MinElute PCR Purification Kit	Qiagen	Cat# 28004
RNeasy Mini Kit	Qiagen	Cat# 74104
AMPure XP beads	Beckman Coulter	Cat# A63881
SPRIselect beads	Beckman Coulter	Cat# B23317
Pierce BCA Protein Assay Kits	ThermoFisher Scientific	Cat# 23225
NEBNext Ultra II RNA Library Prep Kit for Illumina	NEB	Cat# E7760
Dynabeads Oligo(dT)25	ThermoFisher Scientific	Cat# 61005
Lipofectamine RNAiMAX Transfection Reagent	ThermoFisher Scientific	Cat# 13778075
NEBNext HiFi 2x PCR Master mix	NEB	Cat# M0541s
RIPA Lysis and Extraction Buffer	ThermoFisher Scientific	Cat# 89900
NE-PER Nuclear and Cytoplasmic Extraction Reagents	ThermoFisher Scientific	Cat# 78833
Clarity Western ECL Substrate	Bio-Rad	Cat# 170-5060
PageRuler Plus Prestained Protein Ladder	ThermoFisher Scientific	Cat# 26619
Illumina Tagment DNA Enzyme and Buffer Kit	Illumina	Cat #20034211

**Deposited data**		

CUT&Tag	This manuscript	GEO: GSE251654
ATAC-seq	This manuscript	GEO: GSE251654
RNA-seq	This manuscript	GEO: GSE251654

**Experimental models: Cell lines**		

MCF-7	ATCC	Cat# HTB-22
T-47D	ATCC	Cat# HTB-133
MCF-7-*TFF1*-MS2	Rodriguez et al., 2019	N/A
MCF-7-*TFF1* enhancer mutant	Rodriguez et al., 2019	N/A

**Oligonucleotides**		

Single-molecule RNA FISH probes	LGC Biosearch Technologies	See Table S3
Single-molecule RNA FISH probes	IDT	See Table S3
ON-TARGETplus Non-targeting siRNAs	Dharmacon	Cat# D-001810-0X
ON-TARGETplus siRNA-NCOA3 (SRC-3)	Dharmacon	Cat# L-003759-00-0005
ON-TARGETplus siRNA-MED1	Dharmacon	Cat# L-004126-00-0005
Universal i5 and i7 primers with unique barcodes	IDT	Nextera XT Index Kit v2 Set A

**Software and algorithms**		

Micro-Manager	Edelstein et al., 2010	RRID:SCR_000415
CellProfiler 3.1.9	Carpenter et al., 2006	https://cellprofiler.org
Localize	Larson et al., 2013	https://github.com/CBIIT/Larson-Lab-CCR-NCI/tree/master
StochasticGene.jl, v. 1.3.5	Trzaskoma et al. 2024	https://github.com/nih-niddk-mbs/StochasticGene.jl
Cutadapt	Martin, 2011	RRID:SCR_011841
Bowtie2	Langmead & Salzberg, 2012	RRID:SCR_016368
Samtools	Li et al., 2009	RRID:SCR_002105
MACS2	Zhang et al., 2008	RRID:SCR_013291
Bedtools	Quinlan and Hall, 2010	RRID:SCR_006646
ggplot2	Wickham, 2016	RRID:SCR_021139
IGV	Robinson et al., 2011	RRID:SCR_011793

### Experimental model and subject details

MCF-7 and T-47D cells (ATCC) were grown in MEM supplemented with 10% FBS (Sigma), 2mM Glutamine and 1X Pen/strep (Gibco). smFISH experiments were performed in 12 well plates using cells plated on 18mm No. 1.5 coverslips. Cells were plated on coverslips and allowed to recover for 2 days post plating before being hormone depleted. Cells were hormone depleted by washing with Phenol free MEM supplemented with 10% Charcoal/Dextran Treated FBS (Sigma), 2mM Glutamine and 1X Pen/strep. Cells were returned to the incubator for 1h. This step was performed again. This procedure for hormone depletion was followed for all subsequent experiments. After 2 days of hormone depletion, cells were induced with ligand. For the dose response and steady state experiments, cells were induced for 2days, other experiments were performed as described.

For live-cell *TFF1-MS2* imaging MCF-7 cells were plated on glass bottom (Nunc) dishes and allowed to recover for multiple days prior to hormone depletion. Cells were hormone depleted for 2 days prior to addition of ligand 8 h before imaging.

For ICI experiments cells were hormone depleted for 2 days prior to treatment with ligands or ICI (Sigma). Cells were treated with saturating concentrations of E2 or BPA, additionally cells were cotreated with saturating concentrations of E2 or BPA and 100nM ICI.

For CUT&Tag and ATAC-seq MCF-7 cells were plated in 10cm dishes and allowed to recover for 2 days. Cells were subsequently hormone depleted for 2 days prior to induction with ligand. 8 h after induction with ligand nuclei were isolated for CUT&Tag or ATAC-seq.

Cells were plated for washout experiments and allowed to recover for 2 days. Cells were then hormone depleted for 2 days prior to induction with the primary ligand for 8h. Cells were then briefly rinsed with hormone depleted media before induction with the final ligand for 4h.

siRNA experiments were performed by allowing cells to recover for 2 days after plating before hormone depletion. 24h after hormone depletion cells were transfected with siRNA smart pools (Dharmacon) using Lipofectamine RNAiMAX Transfection Reagent and the manufacturers protocol. 24h after transfection cells were induced with ligand for an additional 24h prior to fixation or harvesting for other analysis.

### Method details

#### Single-molecule fluorescence *in situ* hybridization (smFISH)

The *TFF1* exon probe set (16 probes) and intron probe set (79 probes) were previously described.^16^ The *GREB1* intron and exon probe sets (48 probes each) and *EGR3* exon probe set (45 probes) were designed using Stellaris Probe Designer (https://www.biosearchtech.com/stellaris-designer) using a masking level of 5, oligo length of 20, minimum spacing of 2 nucleotides. *TFF1* and *GREB1* exon probe sets were ordered from Biosearch Stellaris in Quasar 570. *TFF1* and *GREB1* intron and *EGR3* exon probe sets were ordered from Biosearch Stellaris in Quasar 670. 3 hybridization chain reaction (HCR) doublets were designed to the intron of *EGR3*. Briefly, Primer3 was used to generate candidate probes. HCR initiator doublets, probes exactly 2bp apart, were selected using a custom python script. HCR initiator probes and fluorescently labeled hairpins were ordered from Integrated DNA Technologies (IDT). All probe sequences are available in Table S3.

We used the Stellaris smFISH protocol as previously described.^16^ Briefly, cells were fixed with 4% PFA for 10 min, washed with PBS, then permeabilized with 70% ethanol overnight at 4°C. Probes were hybridized at 37°C following the Stellaris RNA FISH adherent mammalian cell protocol. Finally, coverslips were mounted using Prolong Gold with DAPI and allowed to dry shielded from light for 24h before imaging.

For smFISH using HCR probe sets sequential HCR and Stellaris smFISH was performed as previously described.^59^ Briefly, HCR FISH was performed first according to the protocol described in^60^ with minor modifications. Probe wash buffer and hybridization buffers both lacked citric acid. Initiator probes were hybridized over night at 37°C and signal amplification was performed at room temperature for 90 min. Following amplification cells were washed 5 times in 5x SSCT before proceeding to the adherent mammalian cell Stellaris RNA FISH Protocol described above.

#### Microscopy

smFISH images were acquired using a custom microscope built on an ASI (www.asiimaging.com/) Rapid Automated Modular Microscope System (RAMM) base fitted with an ASI MS-2000 Small XY stage, a Hamamatsu ORCA-Flash4 V3 CMOS camera (https://www.hamamatsu.com/, C13440-20CU), an ASI High Speed Filter Wheel (FW-1000), ASI MS-2000 Small XY stage, and a Zeiss C-Apochromat 40x/1.20 NA UV-VIS-IR objective. DAPI, Quasar 570, Quasar 670, or Cy5 were excited using a Lumencore SpectraX (https://lumencor.com/) with violet, green, and red filters. The microscope was controlled using Micro-Manager^61^ and twenty-five fields of view consisting of a 10-micron z stack with 0.5 micron intervals.

Live-cell imaging was performed using a Andor Dragonfly Spinning Disk Confocal (https://andor.oxinst.com/) using 37°C incubation and 5% CO_2_. Images were acquired using 488nm excitation, on a Nikon CFI Apo TIRF 60× objective with a pinhole size of 40μm. 7 z planes were acquired with 0.8 micron interval every 100s for 512 frames totaling 14.2h. Analysis was performed using maximum intensity projections.

#### Image analysis

Analysis of smFISH data was performed on maximum intensity projections using a custom python script. Briefly smFISH spots were called by fitting a 2D Gaussian mask and performing local background subtraction. Masks of the cytoplasm and nuclei were generated using CellProfiler.^62^ Transcription sites (TS) were identified in the nuclear mask by intron and exon smFISH spots with a Euclidean distance less than or equal to 5 pixels. Number of nascent transcripts was determined on a per cell basis by extracting the intensity of the exon signal at the identified TS and dividing by median intensity of at least 5 exon smFISH spots detected in the cytoplasmic mask.^16^ Thus, non-productive bursts are not considered in this calculation as both cytoplasmic RNA and exon signal at the TS is required for this calculation.

Analysis of *TFF1-MS2* active and inactive durations was determined using custom IDL software, Localize, as previously described.^16^^,^^63^ Briefly, individual nuclei were tracked over all 512 frames using TrackMate^64^ and the center coordinates of each nuclei was passed to a custom python script which generated a cropped time series for each nuclei. These cropped time series were used by Localize to segment active TSs by fitting a 2D Gaussian mask and performing local background subtraction. Traces were generated by using the track function in Localize which extracts the TS intensity and the background intensity for each frame in between called TS. Tracks were manually inspected to ensure different alleles in the same nuclei were not being tracked. Tracks were then analyzed in python by a Hidden Markov Model (HMM) algorithm to detect active TS and converted to a binary trace denoting frames where *TFF1-MS2* as active and inactive. Finally, cumulative distributions of active and inactive durations were compiled from many traces.

The fraction of active cells was calculated by calling active TS using deepBlink^65^ to analyze each cropped nuclei time series extracted using TrackMate. For each cell the first frame where a TS was detected using deepBlink was recorded, and the cell was considered active. The fraction of active cells was determined by dividing the number of active cells by the total number of tracked cells for each individual frame.

#### Cleavage under targets and tagmentation (CUT&Tag)

CUT&Tag was performed following the Bench top CUT&Tag V.3 protocol.^66^ Briefly, we used 100K nuclei isolated from E2, BPA, or vehicle treated MCF-7 cells. 10 μL BioMag-Plus Concanavalin (Bangs Laboratories) were used per reaction to immobilize the nuclei. Then we added 1:50 mouse ERα (sc-8005, Santa Cruz), 1:25 rabbit FOXA1 (#53528, Cell Signaling Technology), 1:50 mouse H3K27ac (#MA5-23516, Invitrogen), 1:40 Rabbit BRG1 (#NB100-2594, Novus Biologicals) or 1:25 rabbit MED1 (#51613, Cell Signaling Technology) antibody and incubated for 2 h. After one hour of secondary antibody incubation 1.25μL of CUTANA pAG-Tn5 adapter complex was used to load the enzyme to the antibody bound regions. One hour of tagmentation at 37°C was followed by DNA extraction using MinElute PCR Purification Kit (Qiagen). Extracted DNA was subjected to PCR amplification using unique primers sets (Nextera XT v2 Full set (N7-S5)). Number of PCR cycles were determined specific to antibodies to prevent overamplification. We amplified ERα, FOXA1, H3K27ac, BRG1 or MED1 with 14, 15, 15, 15 or 17 PCR cycles, respectively. Libraries were size selected using AMPure XP Beads (Beckman Coulter) with 1.3X ratio. We quantified and assessed the quality of the libraries with Qubit Flex and Tapestation and then sequenced the libraries with 50 bp paired-end reads on Illumina high output NovaSeq SP.

#### ATAC-seq

ATAC-seq samples were prepared following the protocol described in^67^ with minor modifications. First 50K nuclei were isolated in technical duplicate per sample. DNA was tagmented for 30 min at 37°C using the Illumina Tagment DNA kit (20034211). Tagmented DNA was cleaned up using the MinElute PCR Purification Kit (Qiagen). The number of PCR cycles used for library amplification was determined by qPCR. Finally, a double-sided size selection was performed using AMPure XP Beads (Beckman Coulter). Libraries quality was assessed using by Tapestation and the technical duplicate of each sample with the most pronounced nucleosome banding was sequenced.

#### CUT&Tag and ATAC-seq analysis

Briefly, CUT&Tag and ATAC-seq reads were processed using pipelined adapted from.^8^ First adaptors were trimmed using Cutadapt and reads were aligned to the hg38 genome assembly using Bowtie2. Samtools was used to sort the reads and deduplication was performed using Picard Tools (http://broadinstitute.github.io/picard). Samtools was also used to separate reads into read lengths of 1–110 bp, nucleosome free, and 150–250 bp, mono-nucleosome reads. Coverage was generated using bamCoverage and RPKM normalization. Peaks were called using MACS2 and filtered to keep peaks that were called in more than one replicate of at least one condition. Peaks were quantified with the featureCounts tool and differential analyses was performed using the DESeq2 R package. Expressed TSS were identified from publicly available short 5′-capped RNA sequencing data (GSE241599: GSM7731537). This was processed similarly to,^46^ however, hg38 and GENCODE v45 were used. Metaplots were made using the predominant TSS identified as the highest expressed TSS associated with the gene of interest.

#### Western blot

Western blot was carried our as described previously.^68^ Cells were collected and protein isolation was performed using Pierce RIPA buffer (#89901, Thermo Fisher Scientific) for FOXA1 protein or NE-PER Nuclear and Cytoplasmic Extraction Reagents (#78833, Thermo Fisher Scientific) for SRC-3 and MED1 proteins. Cellular extracts (50 μg) or nuclear extracts (70 μg) were loaded on SDS 10%-PAGE or 7.5%-PAGE, respectively. Proteins were probes with antibodies specific to FOXA1 (#53528, Cell Signaling Technology), SRC-3 (#21265, Cell Signaling Technology), MED1 (#51613, Cell Signaling Technology), ERα (sc-8005, Santa Cruz) followed by secondary antibodies conjugated with the horseradish peroxidase (Santa Cruz). Membranes were re-probed with primary antibodies after stripping with varying efficiencies depending on the first used antibody. Antibodies specific to Beta actin (#66009-1-Ig, Proteintech) and Histone 3 (H3, #17168-1-AP, Proteintech) were used for loading control of cellular extracts and nuclear extracts, respectively. Protein images were developed using Clarity Western ECL Substrate (#170–5060, Bio-Rad). Images were captured and quantified with ChemiDoc Imaging System (Bio-Rad). PageRuler Plus Prestained Protein Ladder (#26619, Thermo Fisher Scientific) was used as molecular marker.

#### RNA-seq

Cells were induced with E2, BPA, or vehicle for 24 h and then harvested. Total RNA was isolated using RNeasy Mini Kit (Qiagen). The quantity and quality of the RNA samples were assessed by Tapestation. Polyadenylated mRNAs were selected using Dynabeads Oligo(dT)_25_ (Invitrogen #61005) and libraries were prepared with NEBNext Ultra Directional RNA Library Prep Kit for Illumina (NEB #E7760) following manufacturer’s instruction. Libraries were size selected using AMPure XP Beads (Beckman Coulter). Libraries were quantified Qubit Flex and Tapestation and then sequenced with 75 bp single-end reads on Illumina NextSeq high-output.

Reads were filtered so that only those with a mean quality score of 20 or greater were kept. Adapter was trimmed using Cutadapt version 3.7. Reads were aligned to the hg38 genome assembly using STAR version 2.6.0c. Counts were obtained using the featureCounts tool from the Subread package version 1.5.1 with the GENCODE basic gene annotation version 44. Differential expression was quantified using the DESeq2 R package version 1.34.0.

#### Model fitting

The data was fit using the Julia package StochasticGene.jl, v. 1.3.5 available at https://github.com/nih-niddk-mbs/StochasticGene.jl. The Bayesian posteriors of the rates were computed with a Metropolis-Hastings algorithm on a likelihood function constructed from the chemical master equation of the fully stochastic model. The specific model used in the fits had 3 gene states (G = 3), 3 pre-mRNA or (R = 3), no splicing (S = 0), and the reporter was inserted in step 1 (insertstep = 1). The model had reversible transitions between the repressed and transitional G states, and between the transitional and permissive G states. Each R step could be occupied or unoccupied with a possible transition to the first R step when the permissive G state is occupied and the first R step is unoccupied. The data was fitted directly to the smRNA data and the tracked spots. The fit to the smRNA assumed that mRNA is produced by 5 uncoupled alleles. Although, Rodriguez et al. 2019 showed coupling between the alleles, the coupling was ignored to make the problem computational more tractable. Theoretical analysis indicates that the coupling was probably weak enough to not affect the mRNA histogram enough to dramatically alter the fits. The traces were fit using the hidden Markov model forward algorithm, where the discrete time state transition probability between the time frames (100 s) was computed from the continuous time transition rate matrix of the chemical master equation with a forward Kolmogorov equation. The numerical methods were quite efficient, and the posteriors were computed using millions of MCMC samples using a few hours of computer time on 16 CPUs per from.

### Quantification and statistical analysis

Statistical details of experiments can be found in the results section, figure legends, and method details. For smFISH, unless otherwise stated, t-tests were performed in R using data from three biological replicates for each condition. Statical tests were performed on CUT&Tag, ATAC-seq and RNA-seq data in R using the DESeq2 package. *p*-values are indicated by astricts and follow this format ∗*p* ≤ 0.05 ∗∗*p* ≤ 0.01 ∗∗∗*p* ≤ 0.001. Data is represented at the mean and standard deviation of 3 independent biological replicates unless otherwise stated.

## Supplemental information

Document S1. Figures S1–S7

Table S1. Parameters extracted from the dose response curves (GraphPad Prism), related to Figure 1

Table S2. Posterior transition rate median and standard deviations and posterior median transition times for TFF1 gene (G) state dynamics extracted from E2 and BPA treated cells, related to Figure 2

Table S3. smFISH probe sequences
