# Supplementary material for: Altered cofactor recruitment and nucleosome dynamics underlie bisphenol A’s impact on ERα-mediated transcriptional bursting
Source: iScience. 2025 Jun 10;28(7):112864. doi: 10.1016/j.isci.2025.112864 (PMC12268693; doi:10.1016/j.isci.2025.112864)
Supplement: Document S1. Figures S1–S7 [file mmc1.pdf]

iScience, Volume *28*

Supplemental information

Altered cofactor recruitment and nucleosome dynamics underlie bisphenol A's impact on ERα-mediated transcriptional bursting

Christopher R. Day, Pelin Yaşar, Gloria Adedoyin, Brian D. Bennett, Carson C. Chow, and Joseph Rodriguez

Supplementary Figure 1

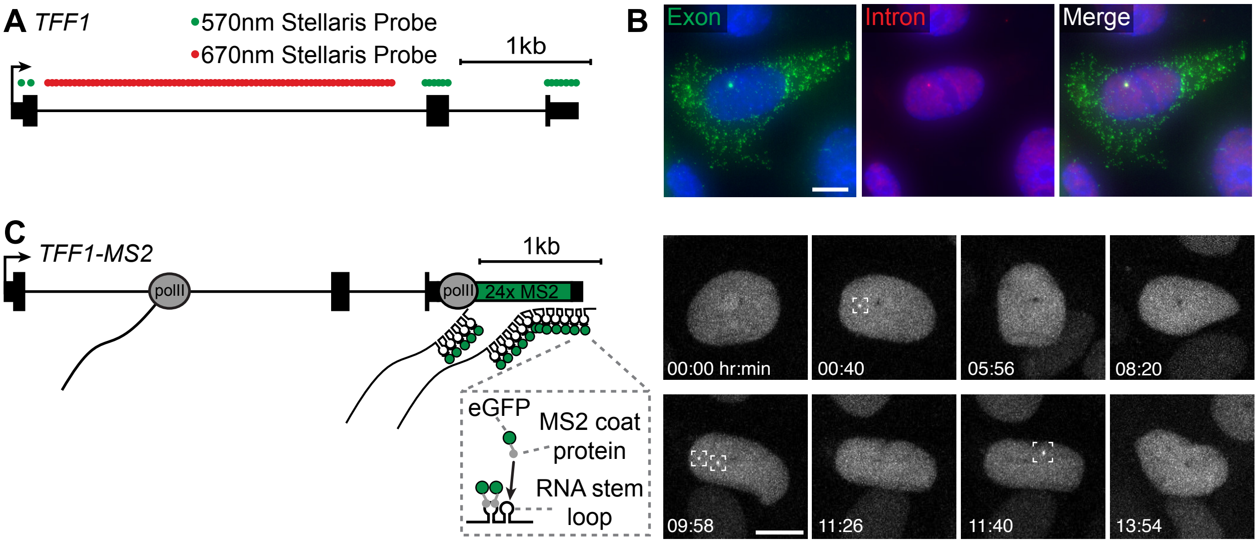

Figure S1. related to Figure 1
(A) Schematic of the *TFF1* transcript and approximate positions of RNA and intron smFISH probes. (B) Representative image of *TFF1* smFISH from MCF-7 cells, RNA visible as green spots, transcriptional bursts are visible where RNA and intron (red) spots colocalize. (C) Schematic of the *TFF1-MS2* system. Representative snapshots of MCF-7 cells with active *TFF1-MS2* bursts outlined with rectangles.

Supplementary Figure 2

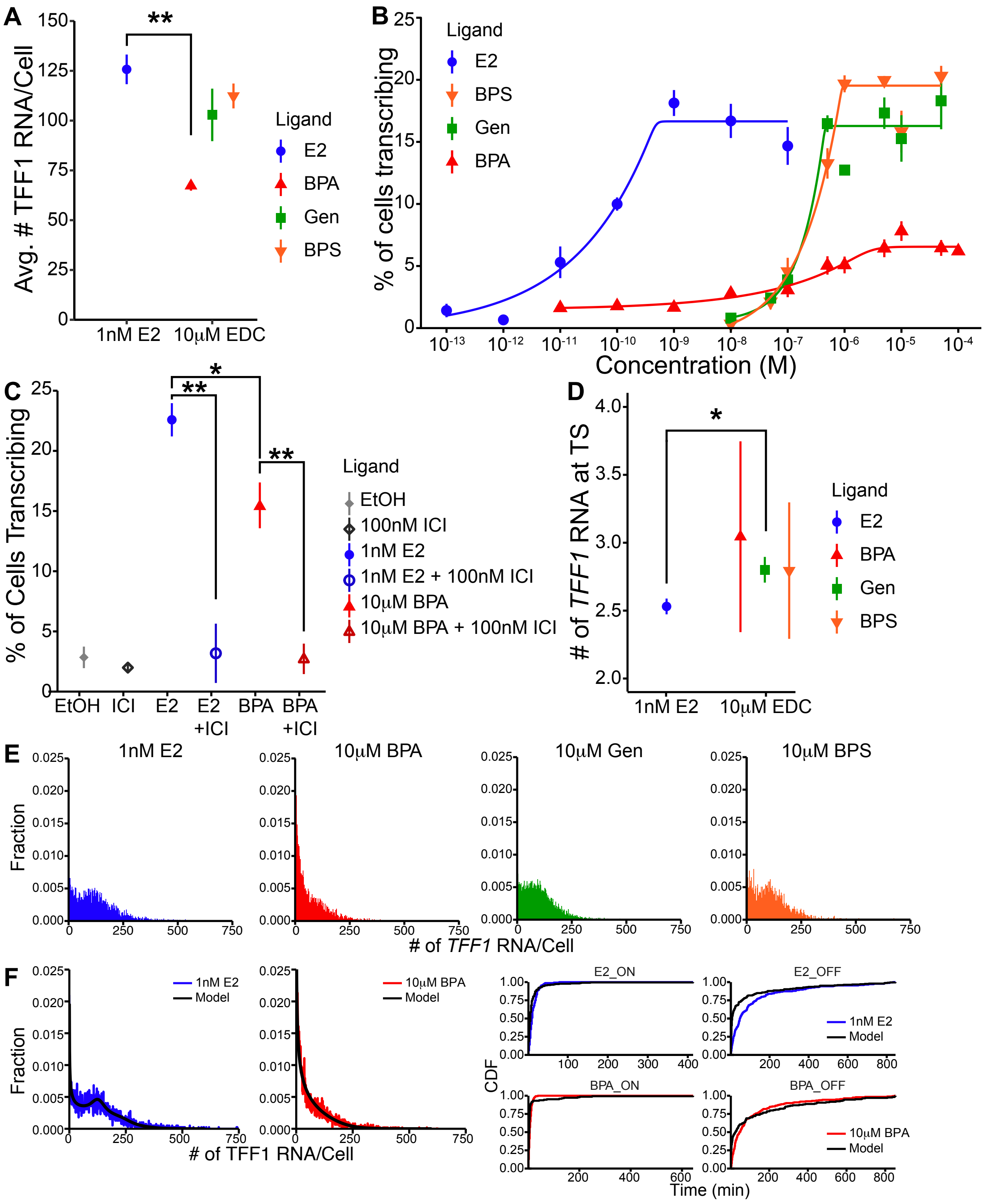

Figure S2. related to Figure 2

(A) smFISH results of the average *TFF1* RNA accumulation in cells treated with E2 or ECDs the mean and standard deviation of 3 replicates is represented. P-values were calculated with t-tests. (B) Dose dependent activation of *TFF1* transcription. Average percent of cells with TS calculated from 3 smFISH replicates in response to E2, BPA, Gen, or BPS fit to a four parameter variable slope function (GraphPad Prism). Error bars represent standard deviation. (C) Average percent of cells with *TFF1* TS. Cells treated with E2 or BPA or cotreated with E2-ICI or BPA-ICI for 24h. P-values were calculated with t-tests and error bars represent SD. (D) Average number of nascent *TFF1* RNA at TS extracted from the smFISH dose response. (E) Histogram of the distribution of *TFF1* RNA per cell extracted from smFISH data extracted from the dose response. (F) Raw smFISH data and live-cell data mathematical model are shown as black lines. * $P \leq 0.05$ ** $P \leq 0.01$

Supplementary Figure 3

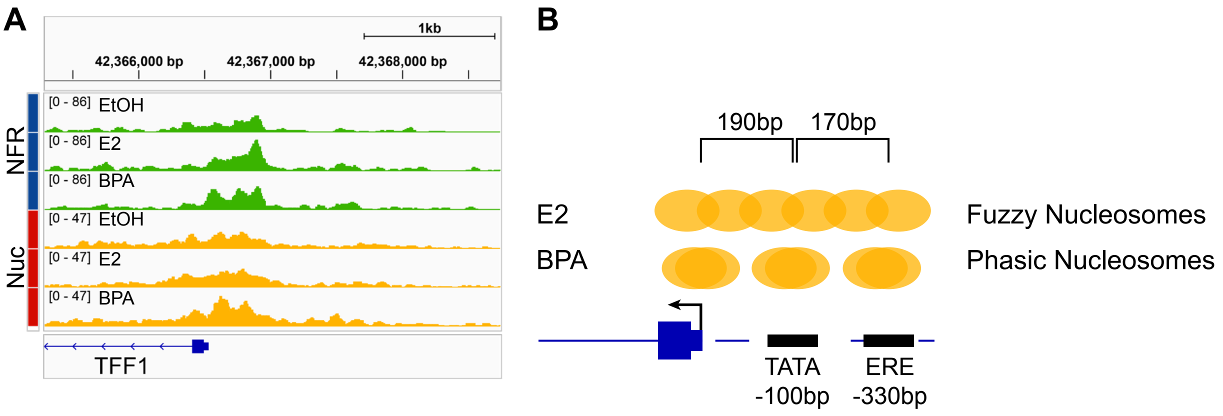

Figure S3. related to Figure 3

(A) Browser tracks of the *TFF1* promoter for nucleosome free ATAC-seq reads (NFR), mono-nucleosome ATAC-seq reads (Nuc). (B) Schematic of the *TFF1* promoter and representations of nucleosome positions based on the ATAC-seq Nuc profiles.

Supplementary Figure 4

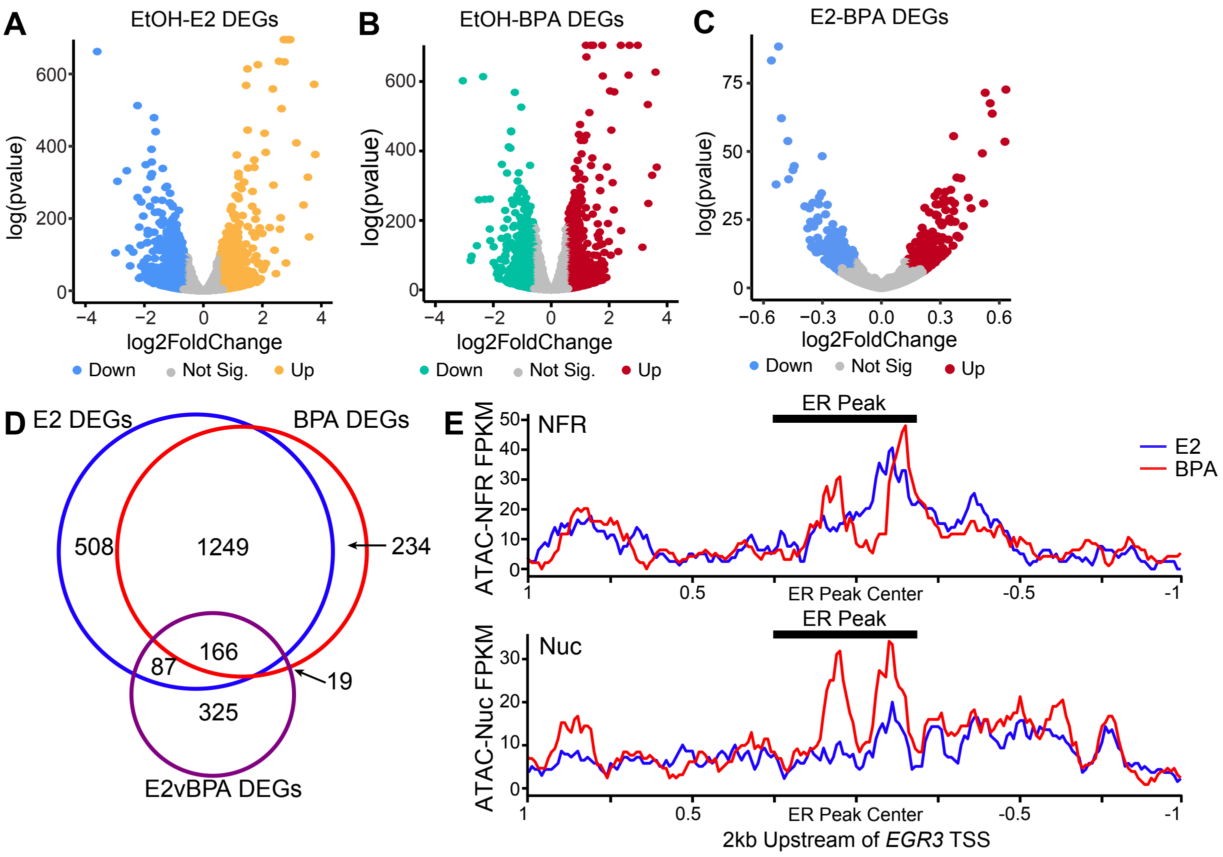

Figure S4. related to Figure 4

(A) Volcano plot of the log2 fold change vs -log(pvalue) from E2/EtOH RNA-seq for all annotated transcripts using DESeq2. Differentially expressed genes (DEGs) identified using thresholds of padj <0.05 and absolute fold change of 1.5. E2 upregulated genes colored yellow (n = 964 genes) and E2 down regulated genes colored bule (n = 1,046 genes). (B) Volcano plot of the log2 fold change vs -log(pvalue) from E2/BPA RNA-seq for all annotated transcripts using DESeq2. DEGs identified using thresholds of with (padj <0.05 and absolute fold change of 1.1). BPA upregulated genes colored red (n = 341 genes) and BPA down regulated genes colored blue (n = 256 genes). (C) RNA-seq Volcano plot of the log2 fold change vs -log(pvalue) from BPA/EtOH for all annotated transcripts using DESeq2. Differentially expressed genes (DEGs) identified using thresholds of padj <0.05 and absolute fold change of 1.1. BPA upregulated genes colored red (n = 831 genes) and BPA down regulated genes colored green (n = 837 genes). (D) Venn-diagram depicting overlap of RNA-seq E2, BPA DEGs and the DEGs identified between E2 or BPA. (E) Line plot of the NFR and Nuc signal extracted at the *EGR3* ER ChIP-seq peak identified in [47].

Supplementary Figure 5

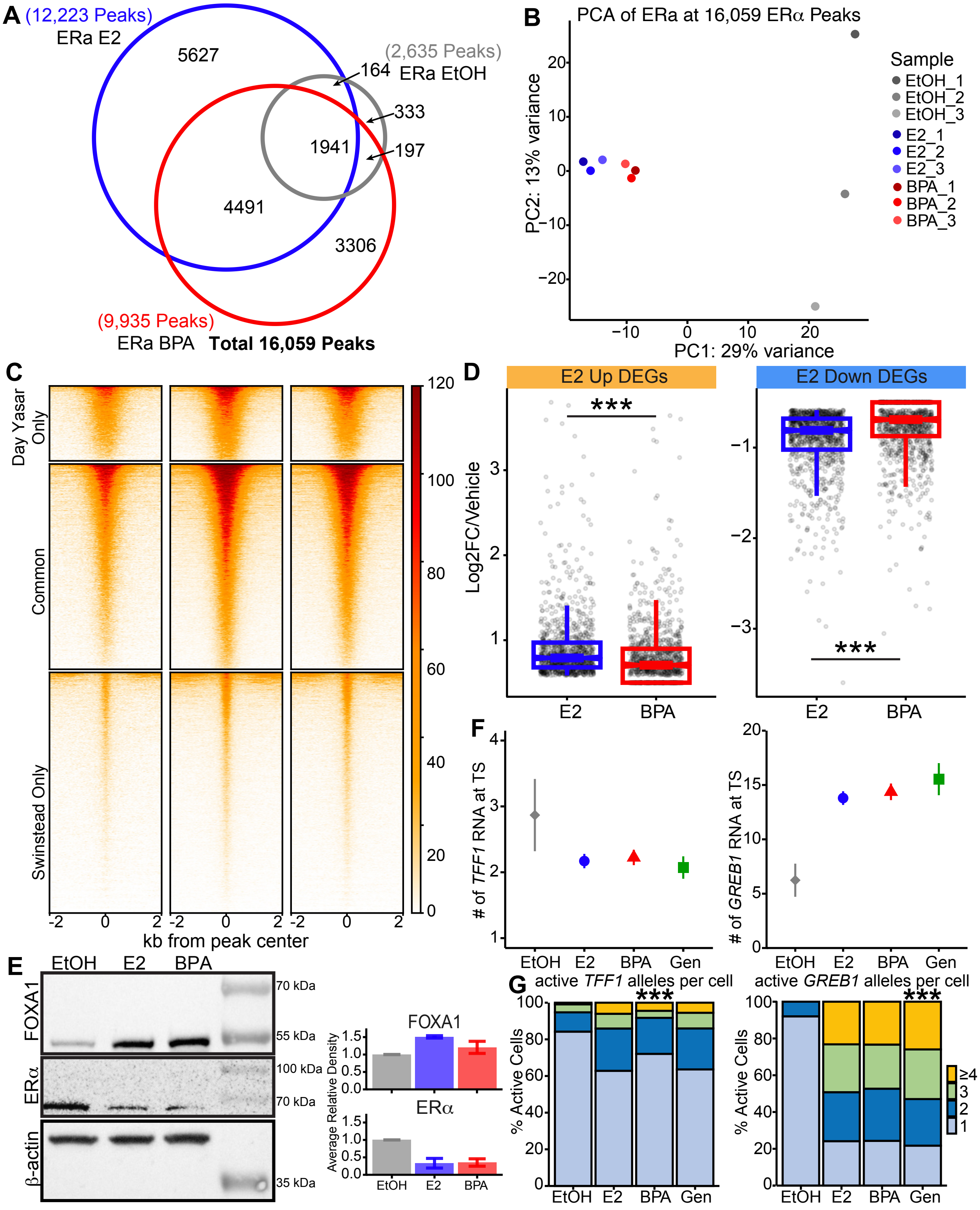

Figure S5. related to Figure 5

(A) Venn-diagram depicting overlap of CUT&Tag peaks identified in cells treated with 1nM E2, 10μM BPA and vehicle for 8h. (B) A plot of PCA of the ERα CUT&Tag results for the 16,059 identified peaks. (C) A heat map of ERα CUT&Tag coverage at identified peaks and previously identified peaks from Swinstead at al. [21] (D) Box plot of the log2 fold change for E2 up and E2 down DEGs calculated using DESeq2 extracted from E2/EtOH RNA-seq and BPA/EtOH RNA-seq samples. (E) WB of total protein using FOXA1, ERα or β-actin specific antibodies from cells treated with vehicle, E2 or BPA and quantification of both FOXA1, ERα protein levels. (F) Average number of nascent *TFF1* and *GREB1* RNA at active TS. Mean and standard deviation calculated from 3 replicates. (G) Frequency distributions of the number of active *TFF1 and GREB1* TS per cell P-values were calculated with Wilcoxon Rank Sum Test. * $P \leq 0.05$ ** $P \leq 0.01$ *** $P \leq 0.001$

Supplementary Figure 6

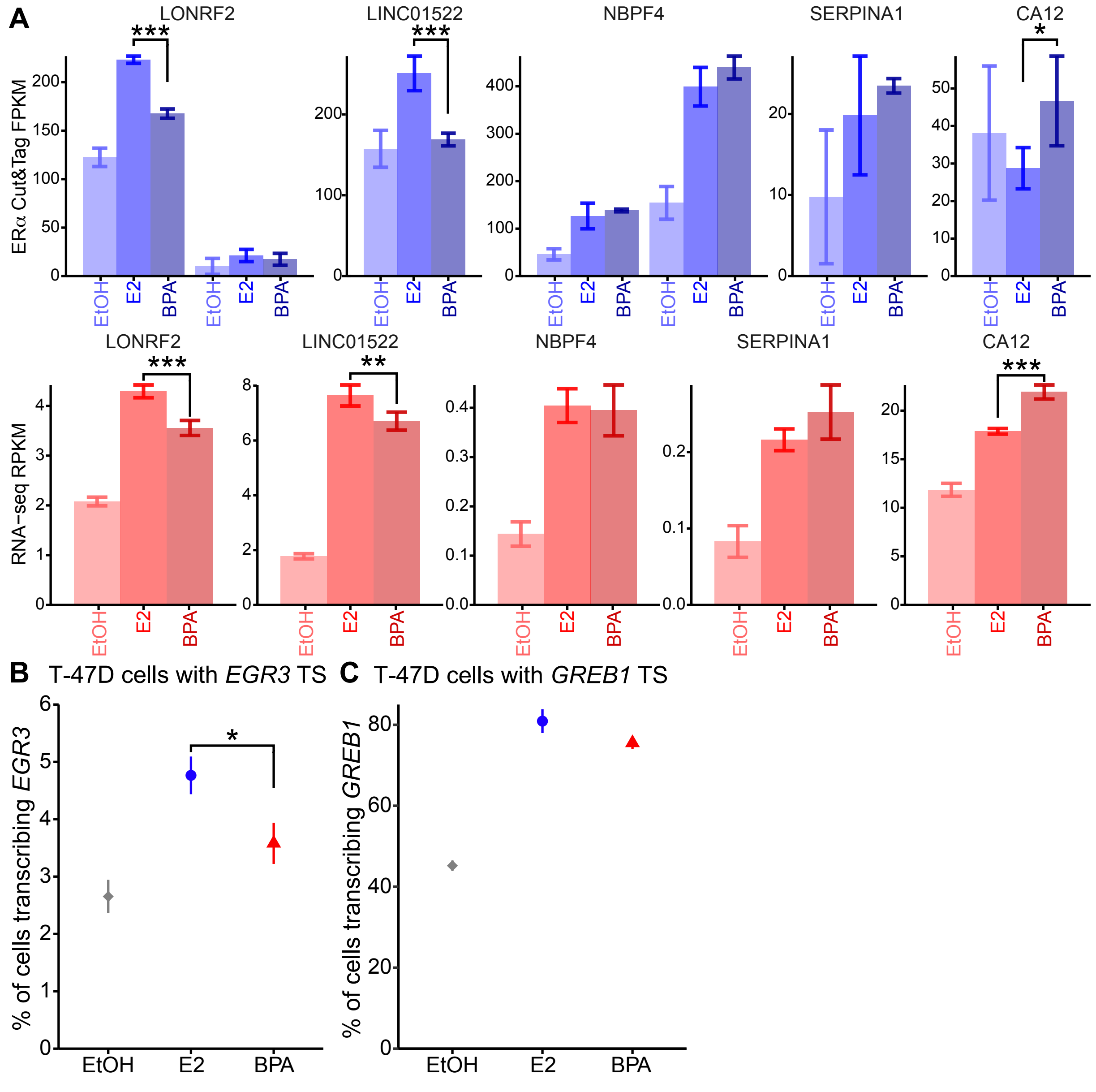

Figure S6. related to Figure 5

Quantification of ERα peaks and quantification of the RNA-seq of the associated gene. ERα peak locations LONRF2: chr2:100290876-100292474, chr2:100321434-100322789. LINC01522: chr20:47989210-47990810. NBPF4: chr1:108234973-108235586, chr1:108237201-108238168. SERPINA1: chr14:94390297-94391164. CA12: chr15:63387437-63388811. (B) Average percent of T-47D cells with *EGR3* TS. (C) Average percent of T-47D cells with *GREB1* TS. P-values were calculated with t-tests and error bars represent SD. * $P \leq 0.05$ ** $P \leq 0.01$ *** $P \leq 0.001$

Supplementary Figure 7

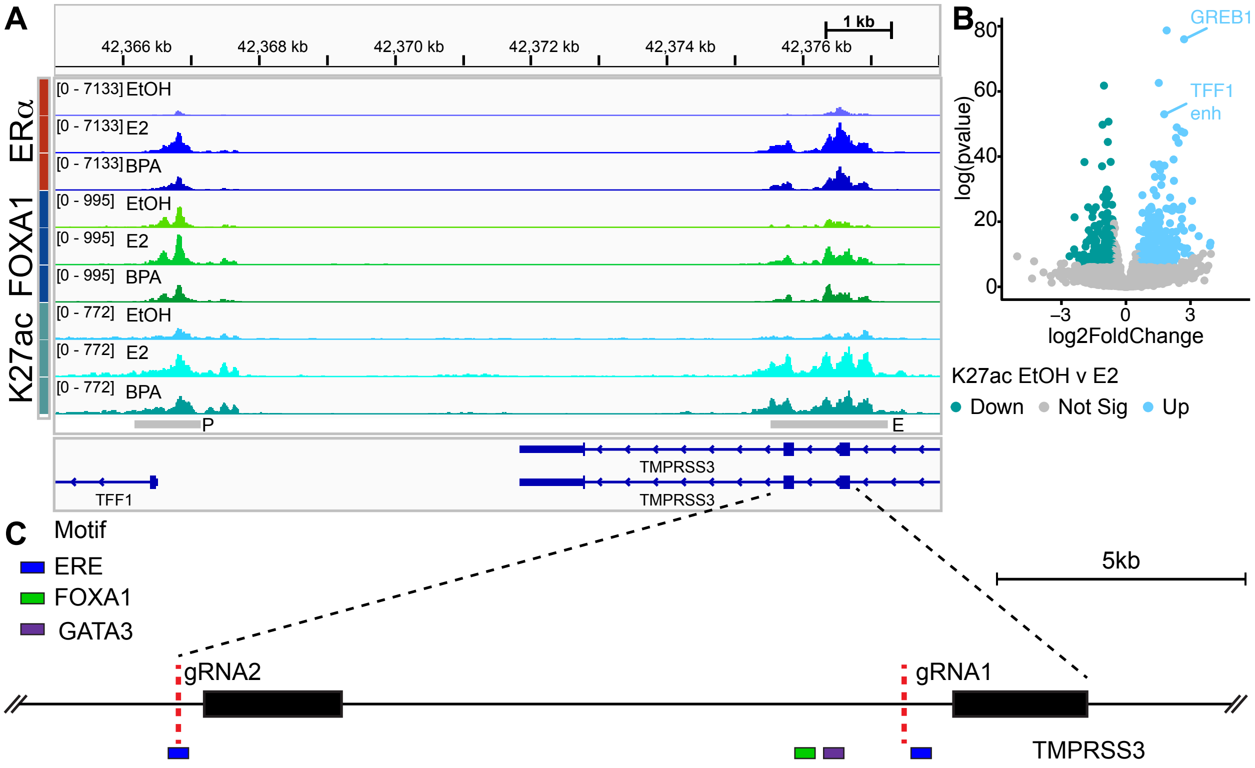

Figure S7. related to Figure 6

(A) Browser tracks of the *TFF1* loci for ERα, FOXA1 and H3K27ac. Promoter (P) and enhancer (E) peaks annotated with gray bars. (B) Volcano plot of the log2 fold change vs -log(pvalue) from E2/EtOH H3K27ac samples using DESeq2. Differential peaks were identified as peaks with (padj <0.05 and absolute fold change of 1.5). E2 increased peak colored cyan (n = 280 peaks) and E2 decreased peaks colored green (n = 354 peaks). (C) Schematic of the *TFF1* enhancer loci depicting validated Estrogen Response Elements (ERE), FOXA1 and GATA3 motifs. Guide RNAs (gRNA) use to generate the *TFF1* Enhancer mutant are annotated by dashed red lines.
